# Supplementary material for: Identification and Characterization of ATOH7-Regulated Target Genes and Pathways in Human Neuroretinal Development
Source: Cells. 2024 Jul 3;13(13):1142. doi: 10.3390/cells13131142 (PMC11240604; doi:10.3390/cells13131142)
Supplement: Supplementary file 1 [file cells-13-01142-s001.zip › Data_S01_Plasmid_Donors/Donor_MUT-GFP.pdf]

```
                                                          10        20        30        40        50        60        70        80        90       100       110
                                                  TGAAGCATTTATCAGGGTTATTGTCTCATGAGCGGATACATATTTGAATGTATTTAGAAAAATAAACAAATAGGGGTTCCGCGCACATTTCCCCGAAAAGTGCCACCTGA
F10-H71500-U519EGG300-4-Regen-seqR.ab1(1>831)  →  TGAAGCATTTATCAGGGTTATTGTCTCATGAGCGGATACATATTTGAATGTATTTAGAAAAATAAACAAATAGGGGTTCCGCGCACATTTCCCCGAAAAGTGCCACCTGA
U519EGG300-4.seq(1>2342)                       →                                                                                                              GA

                                                         120       130       140       150       160       170       180       190       200       210       220
                                                  CGTCCCAATGATTAGGGCGCGCGCCTCCGCGGGGGAAACTTCAACAGACTAATGCTGTAATGTCAGGATTTTGCTGTGCGTCGCCCCCTCCTCCCCGCAGTTCCCAGGGT
F10-H71500-U519EGG300-4-Regen-seqR.ab1(1>831)  →  CGTCCCAATGATTAGGGCGCGCGCCTCCGCGGGGGAAACTTCAACAGACTAATGCTGTAATGTCAGGATTTTGCTGTGCGTCGCCCCCTCCTCCCCGCAGTTCCCAGGGT
U519EGG300-4.seq(1>2342)                       →  CGTCCCAATGATTAGGGCGCGCGCCTCCGCGGGGGAAACTTCAACAGACTAATGCTGTAATGTCAGGATTTTGCTGTGCGTCGCCCCCTCCTCCCCGCAGTTCCCAGGGT

                                                         230       240       250       260       270       280       290       300       310       320       330
                                                  AGGAAGGTGCTGAGATAGAAGCCGAGGGACGCCCGCCCACCTGCCACCACCTGTTCCCTCCTTCAGCTCTTTGCTATAAATTCTCCTCCCTCCAAGATTCATTCTGCACT
F10-H71500-U519EGG300-4-Regen-seqR.ab1(1>831)  →  AGGAAGGTGCTGAGATAGAAGCCGAGGGACGCCCGCCCACCTGCCACCACCTGTTCCCTCCTTCAGCTCTTTGCTATAAATTCTCCTCCCTCCAAGATTCATTCTGCACT
U519EGG300-4.seq(1>2342)                       →  AGGAAGGTGCTGAGATAGAAGCCGAGGGACGCCCGCCCACCTGCCACCACCTGTTCCCTCCTTCAGCTCTTTGCTATAAATTCTCCTCCCTCCAAGATTCATTCTGCACT

                                                         340       350       360       370       380       390       400       410       420       430       440
                                                  CTCCGACAGCTACTGCGCTAAAAGCGCTCCTTCCCTGAGCTTCGGGAAAGAGTTCATCTTCCTGCAAAGGAGTCTCAGGCTTTCCCAGAGGACTTGAAAGGCCTTCCTCG
F10-H71500-U519EGG300-4-Regen-seqR.ab1(1>831)  →  CTCCGACAGCTACTGCGCTAAAAGCGCTCCTTCCCTGAGCTTCGGGAAAGAGTTCATCTTCCTGCAAAGGAGTCTCAGGCTTTCCCAGAGGACTTGAAAGGCCTTCCTCG
U519EGG300-4.seq(1>2342)                       →  CTCCGACAGCTACTGCGCTAAAAGCGCTCCTTCCCTGAGCTTCGGGAAAGAGTTCATCTTCCTGCAAAGGAGTCTCAGGCTTTCCCAGAGGACTTGAAAGGCCTTCCTCG

                                                         450       460       470       480       490       500       510       520       530       540       550
                                                  AACCAGCCACACCAAACTCTGCTGCAGAAGGTTTCCTTCTCTTTTTCAACTTCATGTTGAGAAAATGACTTTCTCTTGAGCATCTCATTTTCCCCTAAATTTGGGCAAGT
F10-H71500-U519EGG300-4-Regen-seqR.ab1(1>831)  →  AACCAGCCACACCAAACTCTGCTGCAGAAGGTTTCCTTCTCTTTTTCAACTTCATGTTGAGAAAATGACTTTCTCTTGAGCATCTCATTTTCCCCTAAATTTGGGCAAGT
U519EGG300-4.seq(1>2342)                       →  AACCAGCCACACCAAACTCTGCTGCAGAAGGTTTCCTTCTCTTTTTCAACTTCATGTTGAGAAAATGACTTTCTCTTGAGCATCTCATTTTCCCCTAAATTTGGGCAAGT
E07-H71500-U519EGG300-4-2-SEQ1F.ab1(1>838)     →                                                                          CTCTTGAGCATCTCATTTTCCCCTAAATTTGGGCAAGT

                                                         560       570       580       590       600       610       620       630       640       650       660
                                                  GAAGAGATATCAGCCTGGTCATCCAGTAGAACAGAAGGCCGAGTCCCGCACTCCCCCACTGTAAACTATTTGATTGCACGTGAGTTGCTTTGTTTATGACTTATTTGCTC
F10-H71500-U519EGG300-4-Regen-seqR.ab1(1>831)  →  GAAGAGATATCAGCCTGGTCATCCAGTAGAACAGAAGGCCGAGTCCCGCACTCCCCCACTGTAAACTATTTGATTGCACGTGAGTTGCTTTGTTTATGACTTATTTGCTC
U519EGG300-4.seq(1>2342)                       →  GAAGAGATATCAGCCTGGTCATCCAGTAGAACAGAAGGCCGAGTCCCGCACTCCCCCACTGTAAACTATTTGATTGCACGTGAGTTGCTTTGTTTATGACTTATTTGCTC
E07-H71500-U519EGG300-4-2-SEQ1F.ab1(1>838)     →  GAAGAGATATCAgCcTgGTCATCCAGTAGAACAGAAGGCCGAGTCCCGCACTCCCCCACTGTAAACTATTTGATTGCACGTGAGTTGCTTTGTTTATGACTTATTTGCTC

                                                         670       680       690       700       710       720       730       740       750       760       770
                                                  AGAAGAGGCACGTTGGGAAGCGGCTCGAGAGACCAGCCCACGCGCAGGTCCTGAGCGGGCGGGCGTGCGAGGTCGGCGACTCGCTGCTTGGGGCCGGGGATGGTGAGCAA
F10-H71500-U519EGG300-4-Regen-seqR.ab1(1>831)  →  AGAAGAGGCACGTTGGGAAGCGGCTCGAGAGACCAGCCCACGCGCAGGTCCTGAGCGGGCGGGCGTGCGAGGTCGGCGACTCGCTGCTTGGGGCCGGGGATGGTGAGCAA
U519EGG300-4.seq(1>2342)                       →  AGAAGAGGCACGTTGGGAAGCGGCTCGAGAGACCAGCCCACGCGCAGGTCCTGAGCGGGCGGGCGTGCGAGGTCGGCGACTCGCTGCTTGGGGCCGGGGatggtgagcaa
E07-H71500-U519EGG300-4-2-SEQ1F.ab1(1>838)     →  AGAAGAGGCACGTTGGGAAGCGGCTCGAGAGACCAGCCCACGCGCAGGTCCTGAGCGGGCGGGCGTGCGAGGTCGGCGACTCGCTGCTTGGGGCCGGGGATGGTGAGCAA

                                                         780       790       800       810       820       830       840       850       860       870       880
                                                  GGGCGAGGAGCTGTTCACCGGGGTGGTGCCCATCCTGGTCGAGCTGGACGGCGACGTAAACGGCCACAAGTTCAGCGTGTCCGGCGAGGGCGAGGGCGATGCCACCTACG
F10-H71500-U519EGG300-4-Regen-seqR.ab1(1>831)  →  GGGCGAGGAGCTGTTCACCGGGGTGGTGCCCATCCTGGTCGAGCTGGACGGCGACGTAAAC
U519EGG300-4.seq(1>2342)                       →  gggcgaggagctgttcaccggggtggtgcccatcctggtcgagctggacggcgacgtaaacggccacaagttcagcgtgtccggcgagggcgagggcgatgccacctacg
E07-H71500-U519EGG300-4-2-SEQ1F.ab1(1>838)     →  GGGCGAGGAGCTGTTCACCGGGGTGGTGCCCATCCTGGTCGAGCTGGACGGCGACGTAAACGGCCACAAGTTCAGCGTGTCCGGCGAGGGCGAGGGCGATGCCACCTACG
```

```
                                                   890       900       910       920       930       940       950       960       970       980       990
                                                GCAAGCTGACCCTGAAGTTCATCTGCACCACCGGCAAGCTGCCCGTGCCCTGGCCCACCCTCGTGACCACCCTGACCTACGGCGTGCAGTGCTTCAGCCGCTACCCCGAC
U519EGG300-4.seq(1>2342)                      → gcaagctgaccctgaagttcatctgcaccaccggcaagctgcccgtgccctggcccaccctcgtgaccaccctgacctacggcgtgcagtgcttcagccgctaccccgac
E07-H71500-U519EGG300-4-2-SEQ1F.ab1(1>838)    → GCAAGCTGACCCTGAAGTTCATCTGCACCACCGGCAAGCTGCCCGTGCCCTGGCCCACCCTCGTGACCACCCTGACCTACGGCGTGCAGTGCTTCAGCCGCTACCCCGAC

                                                  1000      1010      1020      1030      1040      1050      1060      1070      1080      1090      1100
                                                CACATGAAGCAGCACGACTTCTTCAAGTCCGCCATGCCCGAAGGCTACGTCCAGGAGCGCACCATCTTCTTCAAGGACGACGGCAACTACAAGACCCGCGCCGAGGTGAA
U519EGG300-4.seq(1>2342)                      → cacatgaagcagcacgacttcttcaagtccgccatgcccgaaggctacgtccaggagcgcaccatcttcttcaaggacgacggcaactacaagacccgcgccgaggtgaa
E07-H71500-U519EGG300-4-2-SEQ1F.ab1(1>838)    → CACATGAAGCAGCACGACTTCTTCAAGTCCGCCATGCCCGAAGGCTACGTCCAGGAGCGCACCATCTTCTTCAAGGACGACGGCAACTACAAGACCCGCGCCGAGGTGAA

                                                  1110      1120      1130      1140      1150      1160      1170      1180      1190      1200      1210
                                                GTTCGAGGGCGACACCCTGGTGAACCGCATCGAGCTGAAGGGCATCGACTTCAAGGAGGACGGCAACATCCTGGGGCACAAGCTGGAGTACAACTACAACAGCCACAACG
U519EGG300-4.seq(1>2342)                      → gttcgagggcgacaccctggtgaaccgcatcgagctgaagggcatcgacttcaaggaggacggcaacatcctggggcacaagctggagtacaactacaacagccacaacg
E07-H71500-U519EGG300-4-2-SEQ1F.ab1(1>838)    → GTTCGAGGGCGACACCCTGGTGAACCGCATCGAGCTGAAGGGCATCGACTTCAAGGAGGACGGCAACATCCTGGGGCACAAGCTGGAGTACAACTACAACAGCCACAACG
F09-H71500-U519EGG300-4-4-SEQ2F.ab1(1>829)    →                                                                       CTGGGGCACAAGCTGGAGTACAACTACAACAGCCACAACG

                                                  1220      1230      1240      1250      1260      1270      1280      1290      1300      1310      1320
                                                TCTATATCATGGCCGACAAGCAGAAGAACGGCATCAAGGTGAACTTCAAGATCCGCCACAACATCGAGGACGGCAGCGTGCAGCTCGCCGACCACTACCAGCAGAACACC
U519EGG300-4.seq(1>2342)                      → tctatatcatggccgacaagcagaagaacggcatcaaggtgaacttcaagatccgccacaacatcgaggacggcagcgtgcagctcgccgaccactaccagcagaacacc
E07-H71500-U519EGG300-4-2-SEQ1F.ab1(1>838)    → TCTATATCATGGCCGACAAGCAGAAGAACGGCATCAAGGTGAACTTCAAGATCCGCCACAACATCGAGGACGGCAGCGTGCAGCTCGCCGACCACTACCAGCAGAACACC
F09-H71500-U519EGG300-4-4-SEQ2F.ab1(1>829)    → TCTATATCATGGCCGACAAGCAGAAGAACGGCATCAAGGTGAACTTCAAGATCCGCCACAACATCGAGGACGGCAGCGTGCAGCTCGCCGACCACTACCAGCAGAACACC

                                                  1330      1340      1350      1360      1370      1380      1390      1400      1410      1420      1430
                                                CCCATCGGCGACGGCCCCGTGCTGCTGCCCGACAACCACTACCTGAGCACCCAGTCCGCCCTGAGCAAAGACCCCAACGAGAAGCGCGATCACATGGTCCTGCTGGAGTT
U519EGG300-4.seq(1>2342)                      → cccatcggcgacggccccgtgctgctgcccgacaaccactacctgagcacccagtccgccctgagcaaagaccccaacgagaagcgcgatcacatggtcctgctggagtt
E07-H71500-U519EGG300-4-2-SEQ1F.ab1(1>838)    → CCCATCGGCGACGGCCCCGTGCTGCTGCCC
F09-H71500-U519EGG300-4-4-SEQ2F.ab1(1>829)    → CCCATCGGCGACGGCCCCGTGCTGCTGCCCGACAACCACTACCTGAGCACCCAGTCCGCCCTGAGCAAAGACCCCAACGAGAAGCGCGATCACATGGTCCTGCTGGAGTT

                                                  1440      1450      1460      1470      1480      1490      1500      1510      1520      1530      1540
                                                CGTGACCGCCGCCGGGATCACTCTCGGCATGGACGAGCTGTACAAGTAGGGCGCGCGCCTCCGCGGGTGTGGGTGTCCGGCAGCCGCTCCGAGCCTCGGCCCTGCCCCAA
U519EGG300-4.seq(1>2342)                      → cgtgaccgccgccgggatcactctcggcatggacgagctgtacaagTAGGGCGCGCGCCTCCGCGGGTGTGGGTGTCCGGCAGCCGCTCCGAGCCTCGGCCCTGCCCCAA
F09-H71500-U519EGG300-4-4-SEQ2F.ab1(1>829)    → CGTGACCGCCGCCGGGATCACTCTCGGCATGGACGAGCTGTACAAGTAGGGCGCGCGCCTCCGCGGGTGTGGGTGTCCGGCAGCCGCTCCGAGCCTCGGCCCTGCCCCAA

                                                  1550      1560      1570      1580      1590      1600      1610      1620      1630      1640      1650
                                                GTAGCCCAGAAGCCTCCGGCGGCCCAGGATTCTAAGGATGCAATCCTCGAGGAAAATTAGTCGATTCTCAGATTACCTTTATTCGCATCATCAGACCTATGGACGCAATC
U519EGG300-4.seq(1>2342)                      → GTAGCCCAGAAGCCTCCGGCGGCCCAGGATTCTAAGGATGCAATCCTCGAGGAAAATTAGTCGATTCTCAGATTACCTTTATTCGCATCATCAGACCTATGGACGCAATC
F09-H71500-U519EGG300-4-4-SEQ2F.ab1(1>829)    → GTAGCCCAGAAGCCTCCGGCGGCCCAGGATTCTAAGGATGCAATCCTCGAGGAAAATTAGTCGATTCTCAGATTACCTTTATTCGCATCATCAGACCTATGGACGCAATC

                                                  1660      1670      1680      1690      1700      1710      1720      1730      1740      1750      1760
                                                ATTTAATTGCCTTTCTTTTCCCCTCCTCCTTTGTATTTTGTAGATTTCATTAATGGATCTTGTGAATGGGTTGATTGCTGTGAAAATAATGCCCCCTTTCCCCTTTTCTG
U519EGG300-4.seq(1>2342)                      → ATTTAATTGCCTTTCTTTTCCCCTCCTCCTTTGTATTTTGTAGATTTCATTAATGGATCTTGTGAATGGGTTGATTGCTGTGAAAATAATGCCCCCTTTCCCCTTTTCTG
F09-H71500-U519EGG300-4-4-SEQ2F.ab1(1>829)    → ATTTAATTGCCTTTCTTTTCCCCTCCTCCTTTGTATTTTGTAGATTTCATTAATGGATCTTGTGAATGGGTTGATTGCTGTGAAAATAATGCCCCCTTTCCCCTTTTCTG
```

```
                                                      1770      1780      1790      1800      1810      1820      1830      1840      1850      1860      1870
                                                 GGCTACTTTGAGGGAAAACAATCTTAAGAAAAATAGGATTAAGCTATTCTGTTCCAGTCCTCAGAGAAATAATCACTTTCTTAAACTTTGTGAGTTTGTCCTGTTCGGGT
U519EGG300-4.seq(1>2342)                      →  GGCTACTTTGAGGGAAAACAATCTTAAGAAAAATAGGATTAAGCTATTCTGTTCCAGTCCTCAGAGAAATAATCACTTTCTTAAACTTTGTGAGTTTGTCCTGTTCGGGT
F09-H71500-U519EGG300-4-4-SEQ2F.ab1(1>829)    →  GGCTACTTTGAGGGAAAACAATCTTAAGAAAAATAGGATTAAGCTATTCTGTTCCAGTCCTCAGAGAAATAATCACTTTCTTAAACTTTGTGAGTTTGTCCTGTTCGGGT
F11-H71500-U519EGG300-4-4-SEQ3F.ab1(1>826)    →                        CTTAAGAAAAATAGGATTAAGCTATTCTGTTCCAGTCCTCAGAGAAATAATCACTTTCTTAAACTTTGTGAGTTTGTCCTGTTCGGGT

                                                      1880      1890      1900      1910      1920      1930      1940      1950      1960      1970      1980
                                                 GAAGTTACAGTATCCATTACTTGTGTTTGCTCACAACAGAGCTACCTTCCTGTTGTGTAAATGCGTTTTTGCTTTAGTGCATTGTGTGTGCAAGCATGAAGTAGAAACAC
U519EGG300-4.seq(1>2342)                      →  GAAGTTACAGTATCCATTACTTGTGTTTGCTCACAACAGAGCTACCTTCCTGTTGTGTAAATGCGTTTTTGCTTTAGTGCATTGTGTGTGCAAGCATGAAGTAGAAACAC
F09-H71500-U519EGG300-4-4-SEQ2F.ab1(1>829)    →  GAAGTTACAGTATCCATTACTTGTGTTTGCTCACAACAGAGCTACCTTCCTGTTGTGTAAATGCGTTTTTGCTTTAGTGCATTGTGTGTGCAAGCATGAAGTAGAAACAC
F11-H71500-U519EGG300-4-4-SEQ3F.ab1(1>826)    →  GAAGTTACAGTATCCATTACTTGTGTTTGCTCACAACAGAGCTACCTTCCTGTTGTGTAAATGCGTTTTTGCTTTAGTGCATTGTGTGTGCAAGCATGAAGTAGAAACAC
C01-H71500-U519EGG300-4-2-SEQ5F.ab1(1>824)    →                                                            AATGCGTTTTTGCTTTAGTGCATTGTGTGTGCAAGCATGAAGTAGAAACAC

                                                      1990      2000      2010      2020      2030      2040      2050      2060      2070      2080      2090
                                                 TTTTTTTTTCTGGGTACAGTACATGGGTATCGGTGCTCTGTATTTTTTTAAACTGTGTACACATTATTAAAATATACATTTTATAAAATATAAATAAAAACGTGGATTTG
U519EGG300-4.seq(1>2342)                      →  tttttttttCTGGGTACAGTACATGGGTATCGGTGCTCTGTATTTTTTTAAACTGTGTACACATTATTAAAATATACATTTTATAAAATATAAATAAAAACGTGGATTTG
F09-H71500-U519EGG300-4-4-SEQ2F.ab1(1>829)    →  TTTTTTTTTCTGGGTACAG
F11-H71500-U519EGG300-4-4-SEQ3F.ab1(1>826)    →  TTTTTTTTTCTGGGTACAGTACATGGGTATCGGTGCTCTGTATTTTTTTAAACTGTGTACACATTATTAAAATATACATTTTATAAAATATAAATAAAAACGTGGATTTG
C01-H71500-U519EGG300-4-2-SEQ5F.ab1(1>824)    →  TTTTTTTTTCTGGGTACAGTACATGGGTATCGGTGCTCTGTATTTTTTTAAACTGTGTACACATTATTAAAATATACATTTTATAAAATATAAATAAAAACGTGGATTTG

                                                      2100      2110      2120      2130      2140      2150      2160      2170      2180      2190      2200
                                                 TTTTTCATGCCAAGTACCTTGCCAGTTTCCTGATGGCGCTAAAGAACTCTTTGGAGGTGATGATATAGAATGCTTCCCTGAGCAAAGATGTGGAGTGTTTAAACCTAATC
U519EGG300-4.seq(1>2342)                      →  TTTTTCATGCCAAGTACCTTGCCAGTTTCCTGATGGCGCTAAAGAACTCTTTGGAGGTGATGATATAGAATGCTTCCCTGAGCAAAGATGTGGAGTGTTTAAACCTAATC
F11-H71500-U519EGG300-4-4-SEQ3F.ab1(1>826)    →  TTTTTCATGCCAAGTACCTTGCCAGTTTCCTGATGGCGCTAAAGAACTCTTTGGAGGTGATGATATAGAATGCTTCCCTGAGCAAAGATGTGGAGTGTTTAAACCTAATC
C01-H71500-U519EGG300-4-2-SEQ5F.ab1(1>824)    →  TTTTTCATGCCAAGTACCTTGCCAGTTTCCTGATGGCGCTAAAGAACTCTTTGGAGGTGATGATATAGAATGCTTCCCTGAGCAAAGATGTGGAGTGTTTAAACCTAATC

                                                      2210      2220      2230      2240      2250      2260      2270      2280      2290      2300      2310
                                                 CGACCAATAAACGAATGTGCCGCTTTGTAGTAGTTCCTCAAAAAGCTGATGAGAGTACATGTAATTAATTTAAACATTTTCTCTGTCAACTTGTATATTTTTTGTTGATT
U519EGG300-4.seq(1>2342)                      →  CGACCAATAAACGAATGTGCCGCTTTGTAGTAGTTCCTCAAAAAGCTGATGAGAGTACATGTAATTAATTTAAACATTTTCTCTGTCAACTTGTATATTTTTTGTTGATT
F11-H71500-U519EGG300-4-4-SEQ3F.ab1(1>826)    →  CGACCAATAAACGAATGTGCCGCTTTGTAGTAGTTCCTCAAAAAGCTGATGAGAGTACATGTAATTAATTTAAACATTTTCTCTGTCAACTTGTATATTTTTTGTTGATT
C01-H71500-U519EGG300-4-2-SEQ5F.ab1(1>824)    →  CGACCAATAAACGAATGTGCCGCTTTGTAGTAGTTCCTCAAAAAGCTGATGAGAGTACATGTAATTAATTTAAACATTTTCTCTGTCAACTTGTATATTTTTTGTTGATT

                                                      2320      2330      2340      2350      2360      2370      2380      2390      2400      2410      2420
                                                 TTTAAAAAGATTAAAGTGAAATATTTAGCTTTTAAGTAACTAGATACACAGTTCCTCAATATTTTATTATCCACATTAAGCAGATTGCTACTAAGTCCTTACAAGTACCC
U519EGG300-4.seq(1>2342)                      →  TTTAAAAAGATTAAAGTGAAATATTTAGCTTTTAAGTAACTAGATACACAGTTCCTCAATATTTTATTATCCACATTAAGCAGATTGCTACTAAGTCCTTACAAGTACCC
F11-H71500-U519EGG300-4-4-SEQ3F.ab1(1>826)    →  TTTAAAAAGATTAAAGTGAAATATTTAGCTTTTAAGTAACTAGATACACAGTTCCTCAATATTTTATTATCCACATTAAGCAGATTGCTACTAAGTCCTTACAAGTACCC
C01-H71500-U519EGG300-4-2-SEQ5F.ab1(1>824)    →  TTTAAAAAGATTAAAGTGAAATATTTAGCTTTTAAGTAACTAGATACACAGTTCCTCAATATTTTATTATCCACATTAAGCAGATTGCTACTAAGTCCTTACAAGTACCC

                                                      2430      2440      2450      2460      2470      2480      2490      2500      2510      2520      2530
                                                 AAATATATGTAGTTATTCTTATATGTATACAGTAATGTATATATACACATATACAGGAATACACTTCTATATTTCTAAAACTAACACAGAAATTAAAAATATTGCCTTTG
U519EGG300-4.seq(1>2342)                      →  AAATATATGTAGTTATTCTTATATGTATAC
F11-H71500-U519EGG300-4-4-SEQ3F.ab1(1>826)    →  AAATATATGTAGTTATTCTTATATGTATACAGTAATGTATATATACACATATACAGGAATACACTTCTATATTTCTAAAACTAACACAGAAATTAAAAATATTGCCTTTG
C01-H71500-U519EGG300-4-2-SEQ5F.ab1(1>824)    →  AAATATATGTAGTTATTCTTATATGTATACAGTAATGTATATATACACATATACAGGAATACACTTCTATATTTCTAAAACTAACACAGAAATTAAAAATATTGCCTTTG
```

```
                                                    2540      2550      2560      2570      2580      2590      2600      2610      2620      2630      2640
                                                GAAGTGATTTCCTTTTCCTCGCTGCTTGGGGCCGGGGATATCGGAAAGAACATGTGAGCAAAAGGCCAGCAAAAGGCCAGGAACCGTAAAAAGGCCGCGTTGCTGGCGTT
F11-H71500-U519EGG300-4-4-SEQ3F.ab1(1>826)  →  GAAGTGATTTCCTTTTCCTCGCTGCTTGGGGCCGGGGATATCGGAAAGAACATGTGAGCAAAAGGCCAGCAAAAGGCC
C01-H71500-U519EGG300-4-2-SEQ5F.ab1(1>824)  →  GAAGTGATTTCCTTTTCCTCGCTGCTTGGGGCCGGGGATATCGGAAAGAACATGTGAGCAAAAGGCCAGCAAAAGGCCAGGAACCGTAAAAAGGCCGCGTTGCTGGCGTT

                                                    2650      2660      2670      2680      2690      2700      2710      2720      2730      2740      2750
                                                TTTCCATAGGCTCCGCCCCCCTGACGAGCATCACAAAAATCGACGCTCAAGTCAGAGGTGGCGAAACCCGACAGGACTATAAAGATACCAGGCGTTTCCCCCTGGAAGCT
C01-H71500-U519EGG300-4-2-SEQ5F.ab1(1>824)  →  TTTCCATAGGCTCCGCCCCCCTGACGAGCATCACAAAAATCGACGCTCAAGTCAGAGGTGGCGAAACCCGACAGGACTATAAAGATACCAGGCGTTTCCCCCTGGAAGCT

                                                CCC
C01-H71500-U519EGG300-4-2-SEQ5F.ab1(1>824)  →  CCC
```
