# Supplementary material for: Identification and Characterization of ATOH7-Regulated Target Genes and Pathways in Human Neuroretinal Development
Source: Cells. 2024 Jul 3;13(13):1142. doi: 10.3390/cells13131142 (PMC11240604; doi:10.3390/cells13131142)
Supplement: Supplementary file 1 [file cells-13-01142-s001.zip › Data_S01_Plasmid_Donors/Donor_WT-GFP.pdf]

```
                                                10        20        30        40        50        60        70        80        90       100       110
                                          CGCACATTTCCCCGAAAAGTGCCACCTGACGTCCCAATGATTAGGGCGCGCGCCTCCGCGGGGGAAACTTCAACAGACTAATGCTGTAATGTCAGGATTTTGCTGTGCGT
D09-V82868-U127PGC030-4-1.8K-SEQF.ab1(1>844)  →  CGCACATTTCCCCGAAAAGTGCCACCTGACGTCCCAATGATTAGGGCGCGCGCCTCCGCGGGGGAAACTTCAACAGACTAATGCTGTAATGTCAGGATTTTGCTGTGCGT
U127PGC030-4.seq(1>2999)                      →                                          TAGGGCGCGCGCCTCCGCGGGGGAAACTTCAACAGACTAATGCTGTAATGTCAGGATTTTGCTGTGCGT

                                               120       130       140       150       160       170       180       190       200       210       220
                                          CGCCCCCTCCTCCCCGCAGTTCCCAGGGTAGGAAGGTGCTGAGATAGAAGCCGAGGGACGCCCGCCCACCTGCCACCACCTGTTCCCTCCTTCAGCTCTTTGCTATAAAT
D09-V82868-U127PGC030-4-1.8K-SEQF.ab1(1>844)  →  CGCCCCCTCCTCCCCGCAGTTCCCAGGGTAGGAAGGTGCTGAGATAGAAGCCGAGGGACGCCCGCCCACCTGCCACCACCTGTTCCCTCCTTCAGCTCTTTGCTATAAAT
U127PGC030-4.seq(1>2999)                      →  CGCCCCCTCCTCCCCGCAGTTCCCAGGGTAGGAAGGTGCTGAGATAGAAGCCGAGGGACGCCCGCCCACCTGCCACCACCTGTTCCCTCCTTCAGCTCTTTGCTATAAAT

                                               230       240       250       260       270       280       290       300       310       320       330
                                          TCTCCTCCCTCCAAGATTCATTCTGCACTCTCCGACAGCTACTGCGCTAAAAGCGCTCCTTCCCTGAGCTTCGGGAAAGAGTTCATCTTCCTGCAAAGGAGTCTCAGGCT
D09-V82868-U127PGC030-4-1.8K-SEQF.ab1(1>844)  →  TCTCCTCCCTCCAAGATTCATTCTGCACTCTCCGACAGCTACTGCGCTAAAAGCGCTCCTTCCCTGAGCTTCGGGAAAGAGTTCATCTTCCTGCAAAGGAGTCTCAGGCT
U127PGC030-4.seq(1>2999)                      →  TCTCCTCCCTCCAAGATTCATTCTGCACTCTCCGACAGCTACTGCGCTAAAAGCGCTCCTTCCCTGAGCTTCGGGAAAGAGTTCATCTTCCTGCAAAGGAGTCTCAGGCT

                                               340       350       360       370       380       390       400       410       420       430       440
                                          TTCCCAGAGGACTTGAAAGGCCTTCCTCGAACCAGCCACACCAAACTCTGCTGCAGAAGGTTTCCTTCTCTTTTTCAACTTCATGTTGAGAAAATGACTTTCTCTTGAGC
D09-V82868-U127PGC030-4-1.8K-SEQF.ab1(1>844)  →  TTCCCAGAGGACTTGAAAGGCCTTCCTCGAACCAGCCACACCAAACTCTGCTGCAGAAGGTTTCCTTCTCTTTTTCAACTTCATGTTGAGAAAATGACTTTCTCTTGAGC
U127PGC030-4.seq(1>2999)                      →  TTCCCAGAGGACTTGAAAGGCCTTCCTCGAACCAGCCACACCAAACTCTGCTGCAGAAGGTTTCCTTCTCTTTTTCAACTTCATGTTGAGAAAATGACTTTCTCTTGAGC

                                               450       460       470       480       490       500       510       520       530       540       550
                                          ATCTCATTTTCCCCTAAATTTGGGCAAGTGAAGAGATATCAGCCTGGTCATCCAGTAGAACAGAAGGCCGAGTCCCGCACTCCCCCACTGTAAACTATTTGATTGCACGT
D09-V82868-U127PGC030-4-1.8K-SEQF.ab1(1>844)  →  ATCTCATTTTCCCCTAAATTTGGGCAAGTGAAGAGATATCAGCCTGGTCATCCAGTAGAACAGAAGGCCGAGTCCCGCACTCCCCCACTGTAAACTATTTGATTGCACGT
U127PGC030-4.seq(1>2999)                      →  ATCTCATTTTCCCCTAAATTTGGGCAAGTGAAGAGATATCAGCCTGGTCATCCAGTAGAACAGAAGGCCGAGTCCCGCACTCCCCCACTGTAAACTATTTGATTGCACGT
D10-V82868-U127PGC030-4-2-SEQ1F.ab1(1>842)    →                                                                                                         TGCaCGT

                                               560       570       580       590       600       610       620       630       640       650       660
                                          GAGTTGCTTTGTTTATGACTTATTTGCTCAGAAGAGGCACGTTGGGAAGCGGCTCGAGAGACCAGCCCACGCGCAGGTCCTGAGCGGGCGGGCGTGCGAGGTCGGCGACT
D09-V82868-U127PGC030-4-1.8K-SEQF.ab1(1>844)  →  GAGTTGCTTTGTTTATGACTTATTTGCTCAGAAGAGGCACGTTGGGAAGCGGCTCGAGAGACCAGCCCACGCGCAGGTCCTGAGCGGGCGGGCGTGCGAGGTCGGCGACT
U127PGC030-4.seq(1>2999)                      →  GAGTTGCTTTGTTTATGACTTATTTGCTCAGAAGAGGCACGTTGGGAAGCGGCTCGAGAGACCAGCCCACGCGCAGGTCCTGAGCGGGCGGGCGTGCGAGGTCGGCGACT
D10-V82868-U127PGC030-4-2-SEQ1F.ab1(1>842)    →  GAGTTGCTTTGTTTATGACTTATTTGCTCAGAAGAGGCACGTTGGGAAGCGGCTCGAGAGACCAGCCCACGCGCAGGTCCTGAGCGGGCGGGCGTGCGAGGTCGGCGACT

                                               670       680       690       700       710       720       730       740       750       760       770
                                          CGCTGCTTGGGGCCGGGGATGAAGTCCTGCAAGCCCAGCGGCCCGCCGGCGGGAGCGCGCGTTGCACCCCCGTGCGCGGGCGGCACCGAGTGCGCGGGCACGTGCGCCGG
D09-V82868-U127PGC030-4-1.8K-SEQF.ab1(1>844)  →  CGCTGCTTGGGGCCGGGGATGAAGTCCTGCAAGCCCAGCGGCCCGCCGGCGGGAGCGCGCGTTGCACCCCCGTGCGCGGGCGGCACCGAGTGCGCGGGCACGTGCGCCGG
U127PGC030-4.seq(1>2999)                      →  CGCTGCTTGGGGCCGGGGATGAAGTCCTGCAAGCCCAGCGGCCCGCCGGCGGGAGCGCGCGTTGCACCCCCGTGCGCGGGCGGCACCGAGTGCGCGGGCACGTGCGCCGG
D10-V82868-U127PGC030-4-2-SEQ1F.ab1(1>842)    →  CGCTGCTTGGGGCCGGGGATGAAGTCCTGCAAGCCCAGCGGCCCGCCGGCGGGAGCGCGCGTTGCACCCCCGTGCGCGGGCGGCACCGAGTGCGCGGGCACGTGCGCCGG

                                               780       790       800       810       820       830       840       850       860       870       880
                                          GGCCGGGCGGCTGGAGAGCGCGGCGCGCAGGCGCCTGGCGGCCAACGCGCGCGAGCGCCGCCGCATGCAGGGGCTCAACACTGCCTTCGACCGCTTACGCAGGGTGGTTC
D09-V82868-U127PGC030-4-1.8K-SEQF.ab1(1>844)  →  GGCCGGGCGGCTGGAGAGCGCGGCGCGCAgGCGCCTGGCGGCCAACGCGCGCGAGCGCCGCCGCATGCAgGGGC
U127PGC030-4.seq(1>2999)                      →  GGCCGGGCGGCTGGAGAGCGCGGCGCGCAGGCGCCTGGCGGCCAACGCGCGCGAGCGCCGCCGCATGCAGGGGCTCAACACTGCCTTCGACCGCTTACGCAGGGTGGTTC
D10-V82868-U127PGC030-4-2-SEQ1F.ab1(1>842)    →  GGCCGGGCGGCTGGAGAGCGCGGCGCGCAGGCGCCTGGCGGCCAACGCGCGCGAGCGCCGCCGCATGCAGGGGCTCAACACTGCCTTCGACCGCTTACGCAGGGTGGTTC
```

```
                                                 890       900       910       920       930       940       950       960       970       980       990
                                             CCCAGTGGGGCCAGGATAAAAAGCTGTCCAAGTACGAGACCCTGCAGATGGCCCTGAGCTACATCATGGCTCTGACCCGGATCCTGGCCGAGGCCGAGCGATTCGGCTCG
U127PGC030-4.seq(1>2999)                  →  CCCAGTGGGGCCAGGATAAAAAGCTGTCCAAGTACGAGACCCTGCAGATGGCCCTGAGCTACATCATGGCTCTGACCCGGATCCTGGCCGAGGCCGAGCGATTCGGCTCG
D10-V82868-U127PGC030-4-2-SEQ1F.ab1(1>842) →  CCCAGTGGGGCCAGGATAAAAAGCTGTCCAAGTACGAGACCCTGCAGATGGCCCTGAGCTACATCATGGCTCTGACCCGGATCCTGGCCGAGGCCGAGCGATTCGGCTCG

                                                1000      1010      1020      1030      1040      1050      1060      1070      1080      1090      1100
                                             GAGCGGGACTGGGTGGGTCTCCACTGTGAGCACTTCGGCCGCGACCACTACCTCCCGTTCCCGGGCGCGAAGCTGCCGGGCGAGAGCGAGCTGTACAGCCAGAGACTCTT
U127PGC030-4.seq(1>2999)                  →  GAGCGGGACTGGGTGGGTCTCCACTGTGAGCACTTCGGCCGCGACCACTACCTCCCGTTCCCGGGCGCGAAGCTGCCGGGCGAGAGCGAGCTGTACAGCCAGAGACTCTT
D10-V82868-U127PGC030-4-2-SEQ1F.ab1(1>842) →  GAGCGGGACTGGGTGGGTCTCCACTGTGAGCACTTCGGCCGCGACCACTACCTCCCGTTCCCGGGCGCGAAGCTGCCGGGCGAGAGCGAGCTGTACAGCCAGAGACTCTT

                                                1110      1120      1130      1140      1150      1160      1170      1180      1190      1200      1210
                                             CGGCTTCCAGCCCGAGCCCTTCCAGATGGCCACCGAGCAGAAACTCATCTCAGAAGAGGATCTGGGATCCGGAGCTACTAATTTCTCCTTGCTTAAGCAAGCTGGTGATG
U127PGC030-4.seq(1>2999)                  →  CGGCTTCCAGCCCGAGCCCTTCCAGATGGCCACCGAGCAGAAACTCATCTCAGAAGAGGATCTGGGATCCGGAGCTACTAATTTCTCCTTGCTTAAGCAAGCTGGTGATG
D10-V82868-U127PGC030-4-2-SEQ1F.ab1(1>842) →  CGGCTTCCAGCCCGAGCCCTTCCAGATGGCCACCGAGCAGAAACTCATCTCAGAAGAGGATCTGGGATCCGGAGCTACTAATTTCTCCTTGCTTAAGCAAGCTGGTGATG
D12-V82868-U127PGC030-4-2-SEQ2F.ab1(1>838) →                                CaCCGAGCAGAaACTCATCTCAGAAGAGGATCTGGGATCCGGAGCTACTAATTTCTCCTTGCTTAAGCAAGCTGGTGATG

                                                1220      1230      1240      1250      1260      1270      1280      1290      1300      1310      1320
                                             TTGAAGAAAATCCTGGTCCTATGGTGAGCAAGGGCGAGGAGCTGTTCACCGGGGTGGTGCCCATCCTGGTCGAGCTGGACGGCGACGTAAACGGCCACAAGTTCAGCGTG
U127PGC030-4.seq(1>2999)                  →  TTGAAGAAAATCCTGGTCCTATGGTGAGCAAGGGCGAGGAGCTGTTCACCGGGGTGGTGCCCATCCTGGTCGAGCTGGACGGCGACGTAAACGGCCACAAGTTCAGCGTG
D10-V82868-U127PGC030-4-2-SEQ1F.ab1(1>842) →  TTGAAGAAAATCCTGGTCCTATGGTGAGCAAGGGCGAGGAGCTGTTCACCGGGGTGGTGCCCATCCTGGTCGAGCTGGACGGCGACGTAAACGGCCACAAGTTCAGCGTG
D12-V82868-U127PGC030-4-2-SEQ2F.ab1(1>838) →  TTGAAGAAAATCCTGGTCCTATGGTGAGCAAGGGCGAGGAGCTGTTCACCGGGGTGGTGCCCATCCTGGTCGAGCTGGACGGCGACGTAAACGGCCACAAGTTCAGCGTG

                                                1330      1340      1350      1360      1370      1380      1390      1400      1410      1420      1430
                                             TCCGGCGAGGGCGAGGGCGATGCCACCTACGGCAAGCTGACCCTGAAGTTCATCTGCACCACCGGCAAGCTGCCCGTGCCCTGGCCCACCCTCGTGACCACCCTGACCTA
U127PGC030-4.seq(1>2999)                  →  TCCGGCGAGGGCGAGGGCGATGCCACCTACGGCAAGCTGACCCTGAAGTTCATCTGCACCACCGGCAAGCTGCCCGTGCCCTGGCCCACCCTCGTGACCACCCTGACCTA
D10-V82868-U127PGC030-4-2-SEQ1F.ab1(1>842) →  TCCGGCGAGGGCGAGGGCGATGCCACCTACGGCAAGCTGACCCTGAAGTTCATCTGCACCACCGG
D12-V82868-U127PGC030-4-2-SEQ2F.ab1(1>838) →  TCCGGCGAGGGCGAGGGCGATGCCACCTACGGCAAGCTGACCCTGAAGTTCATCTGCACCACCGGCAAGCTGCCCGTGCCCTGGCCCACCCTCGTGACCACCCTGACCTA

                                                1440      1450      1460      1470      1480      1490      1500      1510      1520      1530      1540
                                             CGGCGTGCAGTGCTTCAGCCGCTACCCCGACCACATGAAGCAGCACGACTTCTTCAAGTCCGCCATGCCCGAAGGCTACGTCCAGGAGCGCACCATCTTCTTCAAGGACG
U127PGC030-4.seq(1>2999)                  →  CGGCGTGCAGTGCTTCAGCCGCTACCCCGACCACATGAAGCAGCACGACTTCTTCAAGTCCGCCATGCCCGAAGGCTACGTCCAGGAGCGCACCATCTTCTTCAAGGACG
D12-V82868-U127PGC030-4-2-SEQ2F.ab1(1>838) →  CGGCGTGCAGTGCTTCAGCCGCTACCCCGACCACATGAAGCAGCACGACTTCTTCAAGTCCGCCATGCCCGAAGGCTACGTCCAGGAGCGCACCATCTTCTTCAAGGACG

                                                1550      1560      1570      1580      1590      1600      1610      1620      1630      1640      1650
                                             ACGGCAACTACAAGACCCGCGCCGAGGTGAAGTTCGAGGGCGACACCCTGGTGAACCGCATCGAGCTGAAGGGCATCGACTTCAAGGAGGACGGCAACATCCTGGGGCAC
U127PGC030-4.seq(1>2999)                  →  ACGGCAACTACAAGACCCGCGCCGAGGTGAAGTTCGAGGGCGACACCCTGGTGAACCGCATCGAGCTGAAGGGCATCGACTTCAAGGAGGACGGCAACATCCTGGGGCAC
D12-V82868-U127PGC030-4-2-SEQ2F.ab1(1>838) →  ACGGCAACTACAAGACCCGCGCCGAGGTGAAGTTCGAGGGCGACACCCTGGTGAACCGCATCGAGCTGAAGGGCATCGACTTCAAGGAGGACGGCAACATCCTGGGGCAC

                                                1660      1670      1680      1690      1700      1710      1720      1730      1740      1750      1760
                                             AAGCTGGAGTACAACTACAACAGCCACAACGTCTATATCATGGCCGACAAGCAGAAGAACGGCATCAAGGTGAACTTCAAGATCCGCCACAACATCGAGGACGGCAGCGT
U127PGC030-4.seq(1>2999)                  →  AAGCTGGAGTACAACTACAACAGCCACAACGTCTATATCATGGCCGACAAGCAGAAGAACGGCATCAAGGTGAACTTCAAGATCCGCCACAACATCGAGGACGGCAGCGT
D12-V82868-U127PGC030-4-2-SEQ2F.ab1(1>838) →  AAGCTGGAGTACAACTACAACAGCCACAACGTCTATATCATGGCCGACAAGCAGAAGAACGGCATCAAGGTGAACTTCAAGATCCGCCACAACATCGAGGACGGCAGCGT
```

```
                                               1770      1780      1790      1800      1810      1820      1830      1840      1850      1860      1870
                                               GCAGCTCGCCGACCACTACCAGCAGAACACCCCCATCGGCGACGGCCCCGTGCTGCTGCCCGACAACCACTACCTGAGCACCCAGTCCGCCCTGAGCAAAGACCCCAACG
U127PGC030-4.seq(1>2999)                    →  GCAGCTCGCCGACCACTACCAGCAGAACACCCCCATCGGCGACGGCCCCGTGCTGCTGCCCGACAACCACTACCTGAGCACCCAGTCCGCCCTGAGCAAAGACCCCAACG
D12-V82868-U127PGC030-4-2-SEQ2F.ab1(1>838)  →  GCAGCTCGCCGACCACTACCAGCAGAACACCCCCATCGGCGACGGCCCCGTGCTGCTGCCCGACAACCACTACCTGAGCACCCAGTCCGCCCTGAGCAAAGACCCCAACG
E01-V82868-U127PGC030-4-2-SEQ3F.ab1(1>839)  →                                  CCATCGGCGACGGCCCCGTGCTGCTGCCCGACAACCACTACCTGAGCACCCAGTCCgccctGaGCAAAGACCCCAACG
H12-V82868-U127PGC030-4-2-SEQ3F.ab1(1>842)  →                                      CGGCGACGGCCCCGTGCTGCTGCCCGACAACCACTACCTGAGCACCCAGTCCGCCCTGAGCAAAGACCCCAACG

                                               1880      1890      1900      1910      1920      1930      1940      1950      1960      1970      1980
                                               AGAAGCGCGATCACATGGTCCTGCTGGAGTTCGTGACCGCCGCCGGGATCACTCTCGGCATGGACGAGCTGTACAAGTAGGGCGCGCGCCTCCGCGGGTGTGGGTGTCCG
U127PGC030-4.seq(1>2999)                    →  AGAAGCGCGATCACATGGTCCTGCTGGAGTTCGTGACCGCCGCCGGGATCACTCTCGGCATGGACGAGCTGTACAAGTAGGGCGCGCGCCTCCGCGGGTGTGGGTGTCCG
D12-V82868-U127PGC030-4-2-SEQ2F.ab1(1>838)  →  AGAAGCGCGATCACATGGTCCTGCTGGAGTTCGTGACCGCCGCCGGGATCACTCTCGGCATGGACGAGCTGTACAAGTAGGGCGCGCGCCTCCGCGGG
E01-V82868-U127PGC030-4-2-SEQ3F.ab1(1>839)  →  AGAAGCGCGATCACATGGTCCTGCTGGAGTTCGTGACCGCCGCCGGGATCACTCTCGGCATGGACGAGCTGTACAAGTAGGGCGCGCGCCTCCGCGGGTGTGGGTGTCCG
H12-V82868-U127PGC030-4-2-SEQ3F.ab1(1>842)  →  AGAAGCGCGATCACATGGTCCTGCTGGAGTTCGTGACCGCCGCCGGGATCACTCTCGGCATGGACGAGCTGTACAAGTAGGGCGCGCGCCTCCGCGGGTGTGGGTGTCCG

                                               1990      2000      2010      2020      2030      2040      2050      2060      2070      2080      2090
                                               GCAGCCGCTCCGAGCCTCGGCCCTGCCCCAAGTAGCCCAGAAGCCTCCGGCGGCCCAGGATTCTAAGGATGCAATCCTCGAGGAAAATTAGTCGATTCTCAGATTACCTT
U127PGC030-4.seq(1>2999)                    →  GCAGCCGCTCCGAGCCTCGGCCCTGCCCCAAGTAGCCCAGAAGCCTCCGGCGGCCCAGGATTCTAAGGATGCAATCCTCGAGGAAAATTAGTCGATTCTCAGATTACCTT
E01-V82868-U127PGC030-4-2-SEQ3F.ab1(1>839)  →  GCAGCCGCTCCGAGCCTCGGCCCTGCCCCAAGTAGCCCAGAAGCCTCCGGCGGCCCAGGATTCTAAGGATGCAATCCTCGAGGAAAATTAGTCGATTCTCAGATTACCTT
H12-V82868-U127PGC030-4-2-SEQ3F.ab1(1>842)  →  GCAGCCGCTCCGAGCCTCGGCCCTGCCCCAAGTAGCCCAGAAGCCTCCGGCGGCCCAGGATTCTAAGGATGCAATCCTCGAGGAAAATTAGTCGATTCTCAGATTACCTT

                                               2100      2110      2120      2130      2140      2150      2160      2170      2180      2190      2200
                                               TATTCGCATCATCAGACCTATGGACGCAATCATTTAATTGCCTTTCTTTTCCCCTCCTCCTTTGTATTTTGTAGATTTCATTAATGGATCTTGTGAATGGGTTGATTGCT
U127PGC030-4.seq(1>2999)                    →  TATTCGCATCATCAGACCTATGGACGCAATCATTTAATTGCCTTTCTTTTCCCCTCCTCCTTTGTATTTTGTAGATTTCATTAATGGATCTTGTGAATGGGTTGATTGCT
E01-V82868-U127PGC030-4-2-SEQ3F.ab1(1>839)  →  TATTCGCATCATCAGACCTATGGACGCAATCATTTAATTGCCTTTCTTTTCCCCTCCTCCTTTGTATTTTGTAGATTTCATTAATGGATCTTGTGAATGGGTTGATTGCT
H12-V82868-U127PGC030-4-2-SEQ3F.ab1(1>842)  →  TATTCGCATCATCAGACCTATGGACGCAATCATTTAATTGCCTTTCTTTTCCCCTCCTCCTTTGTATTTTGTAGATTTCATTAATGGATCTTGTGAATGGGTTGATTGCT

                                               2210      2220      2230      2240      2250      2260      2270      2280      2290      2300      2310
                                               GTGAAAATAATGCCCCCTTTCCCCTTTTCTGGGCTACTTTGAGGGAAAACAATCTTAAGAAAAATAGGATTAAGCTATTCTGTTCCAGTCCTCAGAGAAATAATCACTTT
U127PGC030-4.seq(1>2999)                    →  GTGAAAATAATGCCCCCTTTCCCCTTTTCTGGGCTACTTTGAGGGAAAACAATCTTAAGAAAAATAGGATTAAGCTATTCTGTTCCAGTCCTCAGAGAAATAATCACTTT
E01-V82868-U127PGC030-4-2-SEQ3F.ab1(1>839)  →  GTGAAAATAATGCCCCCTTTCCCCTTTTCTGGGCTACTTTGAGGGAAAACAATCTTAAGAAAAATAGGATTAAGCTATTCTGTTCCAGTCCTCAGAGAAATAATCACTTT
H12-V82868-U127PGC030-4-2-SEQ3F.ab1(1>842)  →  GTGAAAATAATGCCCCCTTTCCCCTTTTCTGGGCTACTTTGAGGGAAAACAATCTTAAGAAAAATAGGATTAAGCTATTCTGTTCCAGTCCTCAGAGAAATAATCACTTT

                                               2320      2330      2340      2350      2360      2370      2380      2390      2400      2410      2420
                                               CTTAAACTTTGTGAGTTTGTCCTGTTCGGGTGAAGTTACAGTATCCATTACTTGTGTTTGCTCACAACAGAGCTACCTTCCTGTTGTGTAAATGCGTTTTTGCTTTAGTG
U127PGC030-4.seq(1>2999)                    →  CTTAAACTTTGTGAGTTTGTCCTGTTCGGGTGAAGTTACAGTATCCATTACTTGTGTTTGCTCACAACAGAGCTACCTTCCTGTTGTGTAAATGCGTTTTTGCTTTAGTG
E01-V82868-U127PGC030-4-2-SEQ3F.ab1(1>839)  →  CTTAAACTTTGTGAGTTTGTCCTGTTCGGGTGAAGTTACAGTATCCATTACTTGTGTTTGCTCACAACAGAGCTACCTTCCTGTTGTGTAAATGCGTTTTTGCTTTAGTG
H12-V82868-U127PGC030-4-2-SEQ3F.ab1(1>842)  →  CTTAAACTTTGTGAGTTTGTCCTGTTCGGGTGAAGTTACAGTATCCATTACTTGTGTTTGCTCACAACAGAGCTACCTTCCTGTTGTGTAAATGCGTTTTTGCTTTAGTG

                                               2430      2440      2450      2460      2470      2480      2490      2500      2510      2520      2530
                                               CATTGTGTGTGCAAGCATGAAGTAGAAACACTTTTTTTTTCTGGGTACAGTACATGGGTATCGGTGCTCTGTATTTTTTTAAACTGTGTACACATTATTAAAATATACAT
U127PGC030-4.seq(1>2999)                    →  CATTGTGTGTGCAAGCATGAAGTAGAAACACTTTTTTTTTCTGGGTACAGTACATGGGTATCGGTGCTCTGTATTTTTTTAAACTGTGTACACATTATTAAAATATACAT
E01-V82868-U127PGC030-4-2-SEQ3F.ab1(1>839)  →  CATTGTGTGTGCAAGCATGAAGTAGAAACACTTTTTTTTTCTGGGTACAGTACATGGGTATCGGTGCTCTGTATTTTTTTAAACTGTGTACACATTATTAAAATATACAT
H12-V82868-U127PGC030-4-2-SEQ3F.ab1(1>842)  →  CATTGTGTGTGCAAGCATGAAGTAGAAACACTTTTTTTTTCTGGGTACAGTACATGGGTATCGGTGCTCTGTATTTTTTTAAACTGTGTACACATTATTAAAATATACAT
D11-V82868-U127PGC030-4-2-SEQ4F.ab1(1>835)  →                         AGAaACACTTTTTTTTTCTGGGTACAGTACATGGGTATCGGTGCTCTGTATTTTTTTAAACTGTGTACACATTATTAAAATATACAT
```

```
                                          2540      2550      2560      2570      2580      2590      2600      2610      2620      2630      2640
                                          TTTATAAAATATAAATAAAAACGTGGATTTGTTTTTCATGCCAAGTACCTTGCCAGTTTCCTGATGGCGCTAAAGAACTCTTTGGAGGTGATGATATAGAATGCTTCCCT
U127PGC030-4.seq(1>2999)                → TTTATAAAATATAAATAAAAACGTGGATTTGTTTTTCATGCCAAGTACCTTGCCAGTTTCCTGATGGCGCTAAAGAACTCTTTGGAGGTGATGATATAGAATGCTTCCCT
E01-V82868-U127PGC030-4-2-SEQ3F.ab1(1>839) → TTTATAAAATATAAATAAAAACGTGGATTTGTTTTTCATGCCAAGTACCTTGCCAGTTTCCTGATGGCGCTAAAGAACTCTTTGGAGGTGATGATATAGAA
H12-V82868-U127PGC030-4-2-SEQ3F.ab1(1>842) → TTTATAAAATATAAATAAAAACGTGGATTTGTTTTTCATGCCAAGTACCTTGCCAGTTTCCTGATGGCGCTAAAGAACTCTTTGGAGGTGATGATATAGAATGCTTCC
D11-V82868-U127PGC030-4-2-SEQ4F.ab1(1>835) → TTTATAAAATATAAATAAAAACGTGGATTTGTTTTTCATGCCAAGTACCTTGCCAGTTTCCTGATGGCGCTAAAGAACTCTTTGGAGGTGATGATATAGAATGCTTCCCT

                                          2650      2660      2670      2680      2690      2700      2710      2720      2730      2740      2750
                                          GAGCAAAGATGTGGAGTGTTTAAACCTAATCCGACCAATAAACGAATGTGCCGCTTTGTAGTAGTTCCTCAAAAAGCTGATGAGAGTACATGTAATTAATTTAAACATTT
U127PGC030-4.seq(1>2999)                → GAGCAAAGATGTGGAGTGTTTAAACCTAATCCGACCAATAAACGAATGTGCCGCTTTGTAGTAGTTCCTCAAAAAGCTGATGAGAGTACATGTAATTAATTTAAACATTT
D11-V82868-U127PGC030-4-2-SEQ4F.ab1(1>835) → GAGCAAAGATGTGGAGTGTTTAAACCTAATCCGACCAATAAACGAATGTGCCGCTTTGTAGTAGTTCCTCAAAAAGCTGATGAGAGTACATGTAATTAATTTAAACATTT

                                          2760      2770      2780      2790      2800      2810      2820      2830      2840      2850      2860
                                          TCTCTGTCAACTTGTATATTTTTTGTTGATTTTTAAAAAGATTAAAGTGAAATATTTAGCTTTTAAGTAACTAGATACACAGTTCCTCAATATTTTATTATCCACATTAA
U127PGC030-4.seq(1>2999)                → TCTCTGTCAACTTGTATATTTTTTGTTGATTTTTAAAAAGATTAAAGTGAAATATTTAGCTTTTAAGTAACTAGATACACAGTTCCTCAATATTTTATTATCCACATTAA
D11-V82868-U127PGC030-4-2-SEQ4F.ab1(1>835) → TCTCTGTCAACTTGTATATTTTTTGTTGATTTTTAAAAAGATTAAAGTGAAATATTTAGCTTTTAAGTAACTAGATACACAGTTCCTCAATATTTTATTATCCACATTAA

                                          2870      2880      2890      2900      2910      2920      2930      2940      2950      2960      2970
                                          GCAGATTGCTACTAAGTCCTTACAAGTACCCAAATATATGTAGTTATTCTTATATGTATACAGTAATGTATATATACACATATACAGGAATACACTTCTATATTTCTAAA
U127PGC030-4.seq(1>2999)                → GCAGATTGCTACTAAGTCCTTACAAGTACCCAAATATATGTAGTTATTCTTATATGTATACAGTAATGTATATATACACATATACAGGAATACACTTCTATATTTCTAAA
D11-V82868-U127PGC030-4-2-SEQ4F.ab1(1>835) → GCAGATTGCTACTAAGTCCTTACAAGTACCCAAATATATGTAGTTATTCTTATATGTATACAGTAATGTATATATACACATATACAGGAATACACTTCTATATTTCTAAA

                                          2980      2990      3000      3010      3020      3030      3040      3050      3060      3070      3080
                                          ACTAACACAGAAATTAAAAATATTGCCTTTGGAAGTGATTTCCTTTTCCTCGCTGCTTGGGGCCGGGGATATCGGAAAGAACATGTGAGCAAAAGGCCAGCAAAAGGCCA
U127PGC030-4.seq(1>2999)                → ACTAACACAGAAATTAAAAATATTGCCTTTGGAAGTGATTTCCTTTTCCTCGCTGCTTGGGGCCGGGGAT
D11-V82868-U127PGC030-4-2-SEQ4F.ab1(1>835) → ACTAACACAGAAATTAAAAATATTGCCTTTGGAAGTGATTTCCTTTTCCTCGCTGCTTGGGGCCGGGGATATCGGAAAGAACATGTGAGCAAAAGGCCAGCAAAAGGCCA

                                          3090      3100      3110      3120      3130      3140      3150      3160      3170      3180      3190
                                          GGAACCGTAAAAAGGCCGCGTTGCTGGCGTTTTTCCATAGGCTCCGCCCCCCTGACGAGCATCACAAAAATCGACGCTCAAGTCAGAGGTGGCGAAACCCGACAGGACTA
D11-V82868-U127PGC030-4-2-SEQ4F.ab1(1>835) → GGAACCGTAAAAAGGCCGCGTTGCTGGCGTTTTTCCATAGGCTCCGCCCCCCTGACGAGCATCACAAAAATCGACGCTCAAGTCAGAGGTGGCGAAACCCGACAGGACTA

                                          3200      3210      3220      3230      3240      3250      3260      3270
                                          TAAAGATACCAGGCGTTTCCCCCTGGAAGCTCCCTCGTGCGCTCTCCTGTTCCGACCCTGCCGCTTACCGGATACCTGTCCGCCTTTC
D11-V82868-U127PGC030-4-2-SEQ4F.ab1(1>835) → TAAAGATACCAGGCGTTTCCCCCTGgAAGCTCCCTCGTGCGCTCTCCTGTTCCGACCCTGCCGCTTACCGGATACCTGTCCGCCTTTC
```
