# Supplementary material for: Identification and Characterization of ATOH7-Regulated Target Genes and Pathways in Human Neuroretinal Development
Source: Cells. 2024 Jul 3;13(13):1142. doi: 10.3390/cells13131142 (PMC11240604; doi:10.3390/cells13131142)
Supplement: Supplementary file 1 [file cells-13-01142-s001.zip › Supplemental Figures_202406.pptx]

## Slide 1
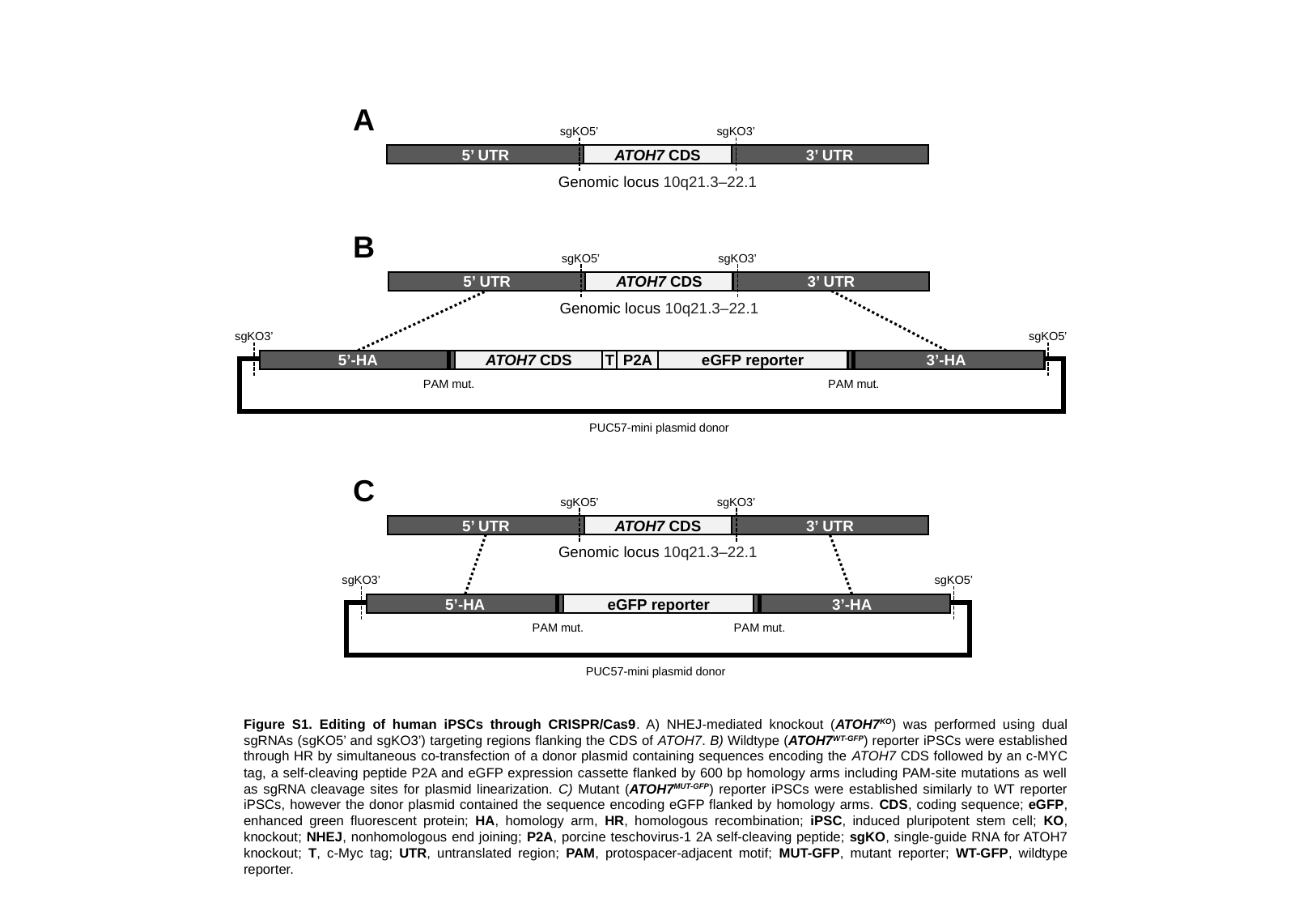

A
sgKO5’
sgKO3’
5’ UTR
ATOH7 CDS
3’ UTR
Genomic locus 10q21.3–22.1
B
sgKO5’
sgKO3’
5’ UTR
ATOH7 CDS
3’ UTR
Genomic locus 10q21.3–22.1
sgKO3’
sgKO5’
T
P2A
5’-HA
ATOH7 CDS
eGFP reporter
3’-HA
PAM mut.
PAM mut.
PUC57-mini plasmid donor
C
sgKO5’
sgKO3’
5’ UTR
ATOH7 CDS
3’ UTR
Genomic locus 10q21.3–22.1
sgKO3’
sgKO5’
5’-HA
eGFP reporter
3’-HA
PAM mut.
PAM mut.
PUC57-mini plasmid donor
Figure S1. Editing of human iPSCs through CRISPR/Cas9. A) NHEJ-mediated knockout (ATOH7KO) was performed using dual sgRNAs (sgKO5’ and sgKO3’) targeting regions flanking the CDS of ATOH7. B) Wildtype (ATOH7WT-GFP) reporter iPSCs were established through HR by simultaneous co-transfection of a donor plasmid containing sequences encoding the ATOH7 CDS followed by an c-MYC tag, a self-cleaving peptide P2A and eGFP expression cassette flanked by 600 bp homology arms including PAM-site mutations as well as sgRNA cleavage sites for plasmid linearization. C) Mutant (ATOH7MUT-GFP) reporter iPSCs were established similarly to WT reporter iPSCs, however the donor plasmid contained the sequence encoding eGFP flanked by homology arms. CDS, coding sequence; eGFP, enhanced green fluorescent protein; HA, homology arm, HR, homologous recombination; iPSC, induced pluripotent stem cell; KO, knockout; NHEJ, nonhomologous end joining; P2A, porcine teschovirus-1 2A self-cleaving peptide; sgKO, single-guide RNA for ATOH7 knockout; T, c-Myc tag; UTR, untranslated region; PAM, protospacer-adjacent motif; MUT-GFP, mutant reporter; WT-GFP, wildtype reporter.

## Slide 2
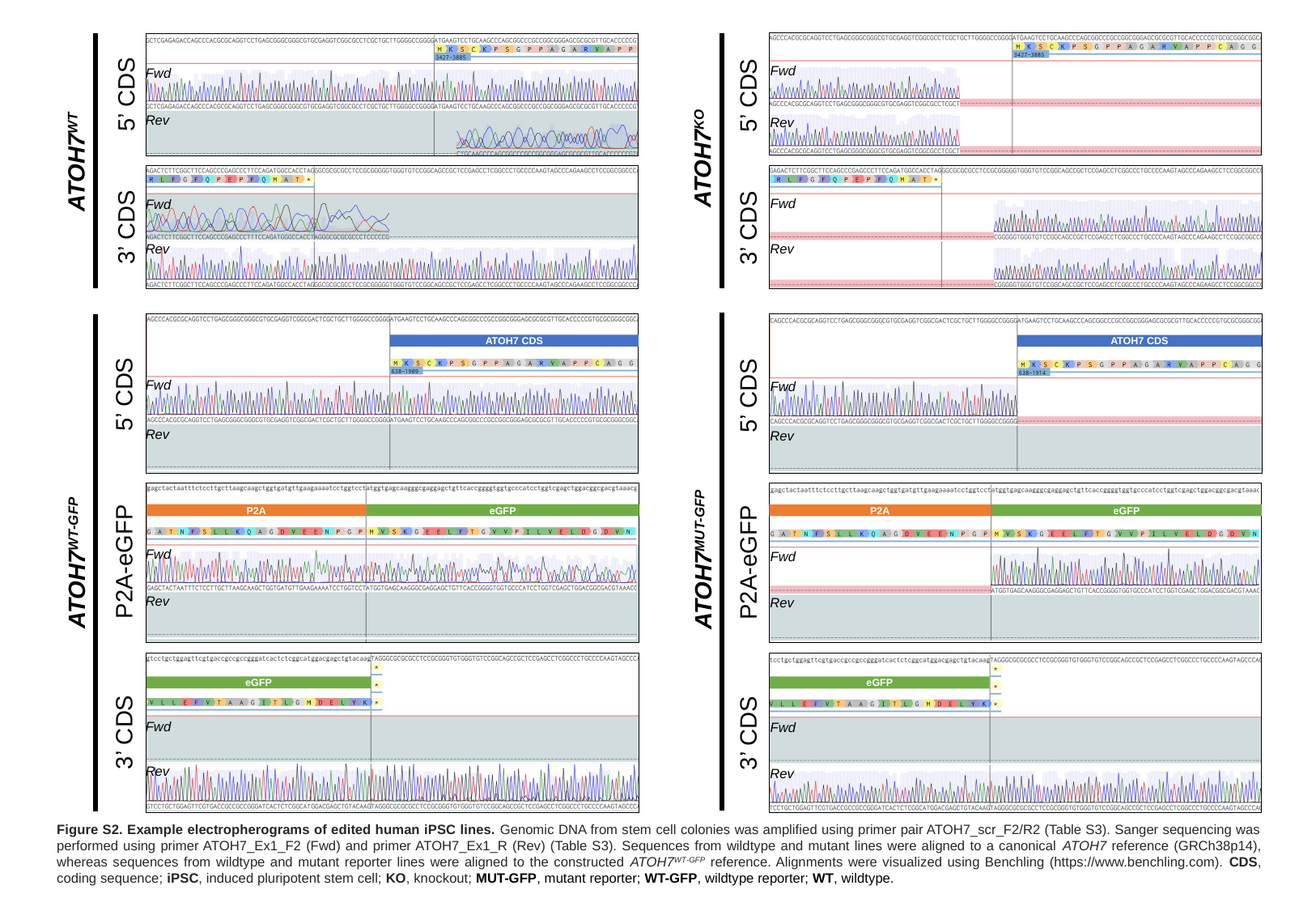

Fwd
Fwd
5’ CDS
5’ CDS
Rev
Rev
ATOH7KO
ATOH7WT
Fwd
Fwd
3’ CDS
3’ CDS
Rev
Rev
ATOH7 CDS
ATOH7 CDS
5’ CDS
5’ CDS
Fwd
Fwd
Rev
Rev
P2A
eGFP
P2A
eGFP
ATOH7MUT-GFP
P2A-eGFP
P2A-eGFP
ATOH7WT-GFP
Fwd
Fwd
Rev
Rev
eGFP
eGFP
3’ CDS
3’ CDS
Fwd
Fwd
Rev
Rev
Figure S2. Example electropherograms of edited human iPSC lines. Genomic DNA from stem cell colonies was amplified using primer pair ATOH7_scr_F2/R2 (Table S3). Sanger sequencing was performed using primer ATOH7_Ex1_F2 (Fwd) and primer ATOH7_Ex1_R (Rev) (Table S3). Sequences from wildtype and mutant lines were aligned to a canonical ATOH7 reference (GRCh38p14), whereas sequences from wildtype and mutant reporter lines were aligned to the constructed ATOH7WT-GFP reference. Alignments were visualized using Benchling (https://www.benchling.com). CDS, coding sequence; iPSC, induced pluripotent stem cell; KO, knockout; MUT-GFP, mutant reporter; WT-GFP, wildtype reporter; WT, wildtype.

## Slide 3
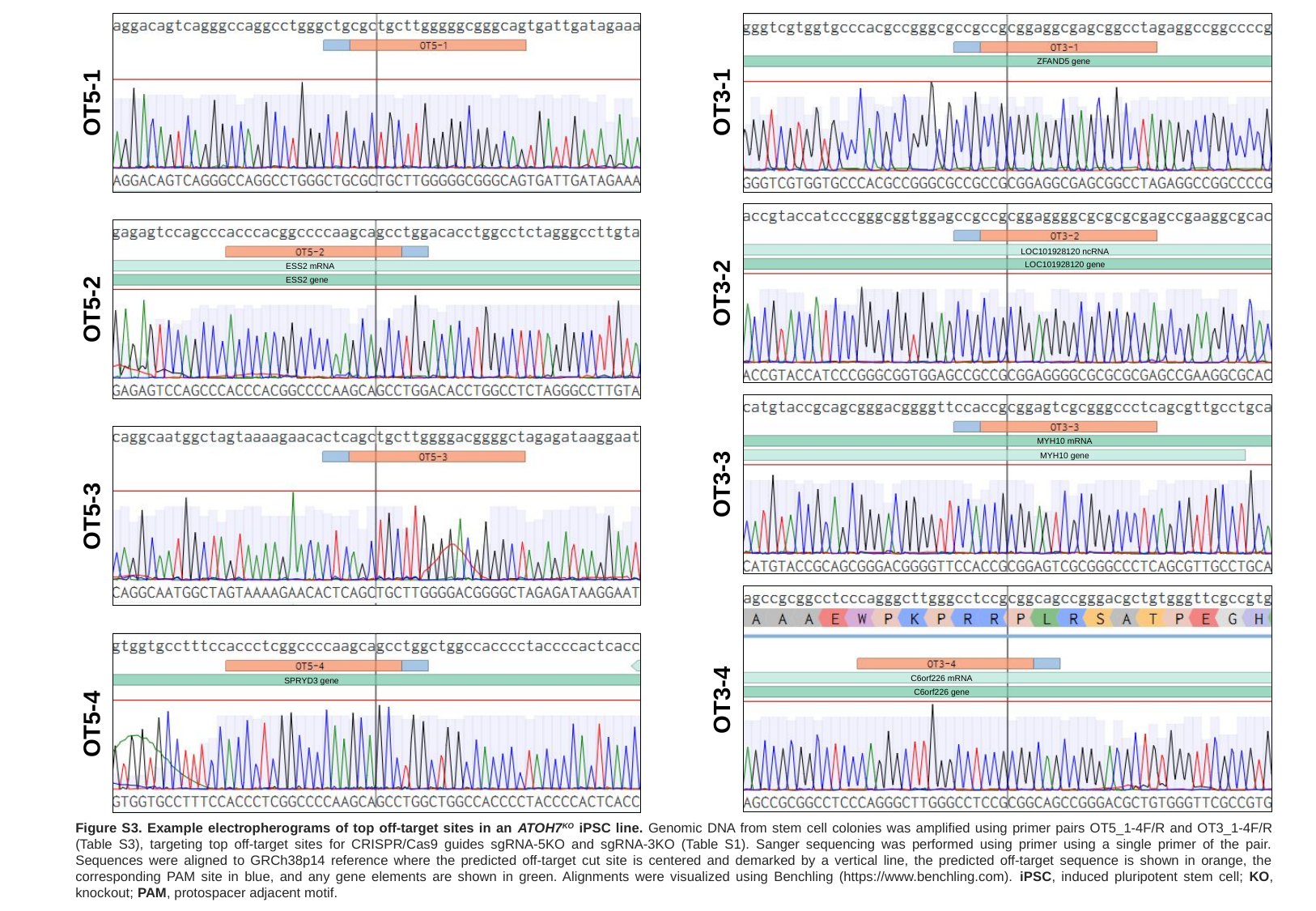

ZFAND5 gene
OT3-1
OT5-1
LOC101928120 ncRNA
LOC101928120 gene
ESS2 mRNA
ESS2 gene
OT3-2
OT5-2
MYH10 mRNA
MYH10 gene
OT3-3
OT5-3
C6orf226 mRNA
SPRYD3 gene
OT3-4
C6orf226 gene
OT5-4
Figure S3. Example electropherograms of top off-target sites in an ATOH7KO iPSC line. Genomic DNA from stem cell colonies was amplified using primer pairs OT5_1-4F/R and OT3_1-4F/R (Table S3), targeting top off-target sites for CRISPR/Cas9 guides sgRNA-5KO and sgRNA-3KO (Table S1). Sanger sequencing was performed using primer using a single primer of the pair. Sequences were aligned to GRCh38p14 reference where the predicted off-target cut site is centered and demarked by a vertical line, the predicted off-target sequence is shown in orange, the corresponding PAM site in blue, and any gene elements are shown in green. Alignments were visualized using Benchling (https://www.benchling.com). iPSC, induced pluripotent stem cell; KO, knockout; PAM, protospacer adjacent motif.

## Slide 4
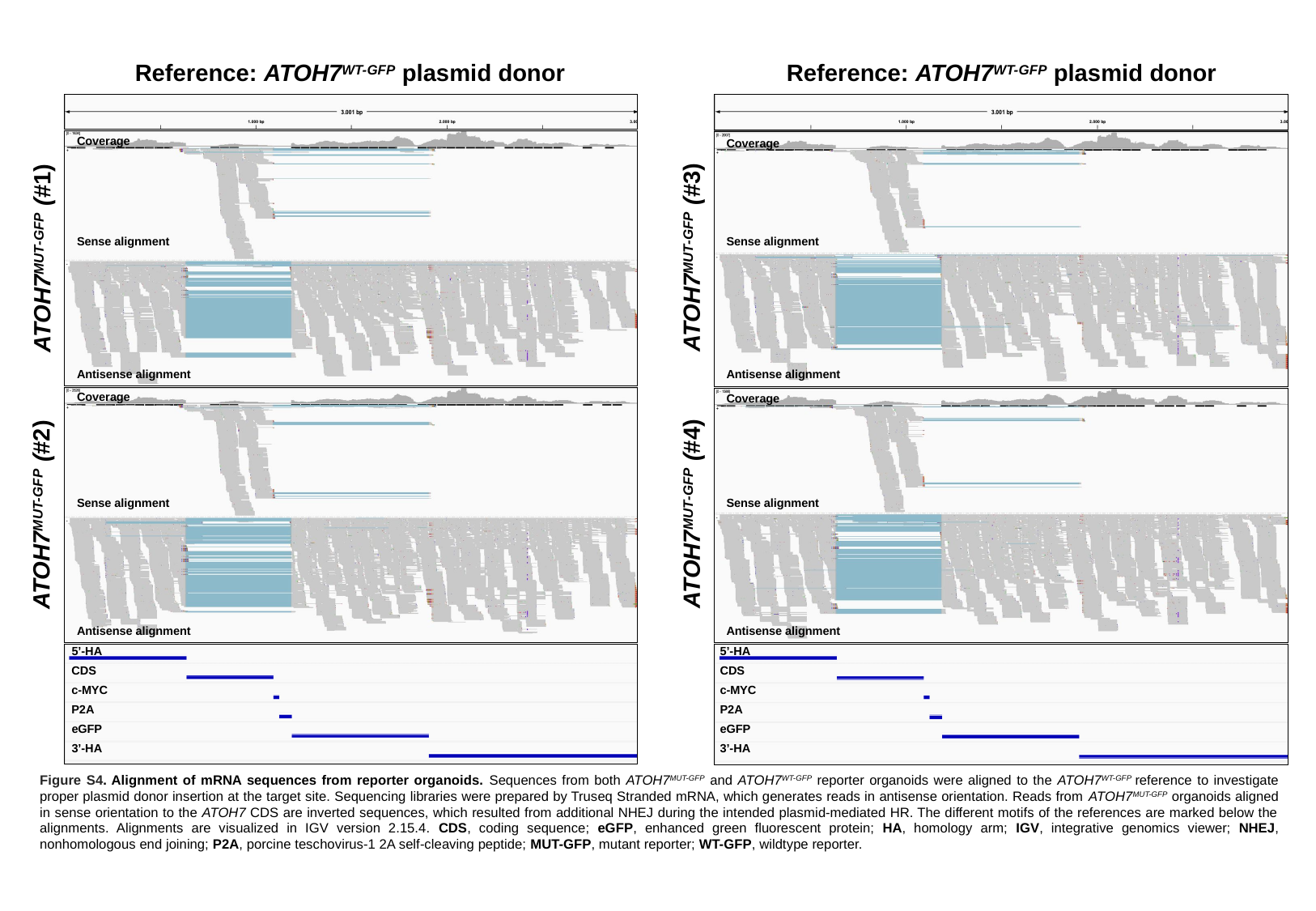

Reference: ATOH7WT-GFP plasmid donor
Reference: ATOH7WT-GFP plasmid donor
Coverage
Coverage
Sense alignment
Sense alignment
ATOH7MUT-GFP (#3)
ATOH7MUT-GFP (#1)
Antisense alignment
Antisense alignment
Coverage
Coverage
Sense alignment
Sense alignment
ATOH7MUT-GFP (#4)
ATOH7MUT-GFP (#2)
Antisense alignment
Antisense alignment
5’-HA
CDS
c-MYC
P2A
eGFP
3’-HA
5’-HA
CDS
c-MYC
P2A
eGFP
3’-HA
Figure S4. Alignment of mRNA sequences from reporter organoids. Sequences from both ATOH7MUT-GFP and ATOH7WT-GFP reporter organoids were aligned to the ATOH7WT-GFP reference to investigate proper plasmid donor insertion at the target site. Sequencing libraries were prepared by Truseq Stranded mRNA, which generates reads in antisense orientation. Reads from ATOH7MUT-GFP organoids aligned in sense orientation to the ATOH7 CDS are inverted sequences, which resulted from additional NHEJ during the intended plasmid-mediated HR. The different motifs of the references are marked below the alignments. Alignments are visualized in IGV version 2.15.4. CDS, coding sequence; eGFP, enhanced green fluorescent protein; HA, homology arm; IGV, integrative genomics viewer; NHEJ, nonhomologous end joining; P2A, porcine teschovirus-1 2A self-cleaving peptide; MUT-GFP, mutant reporter; WT-GFP, wildtype reporter.

## Slide 5
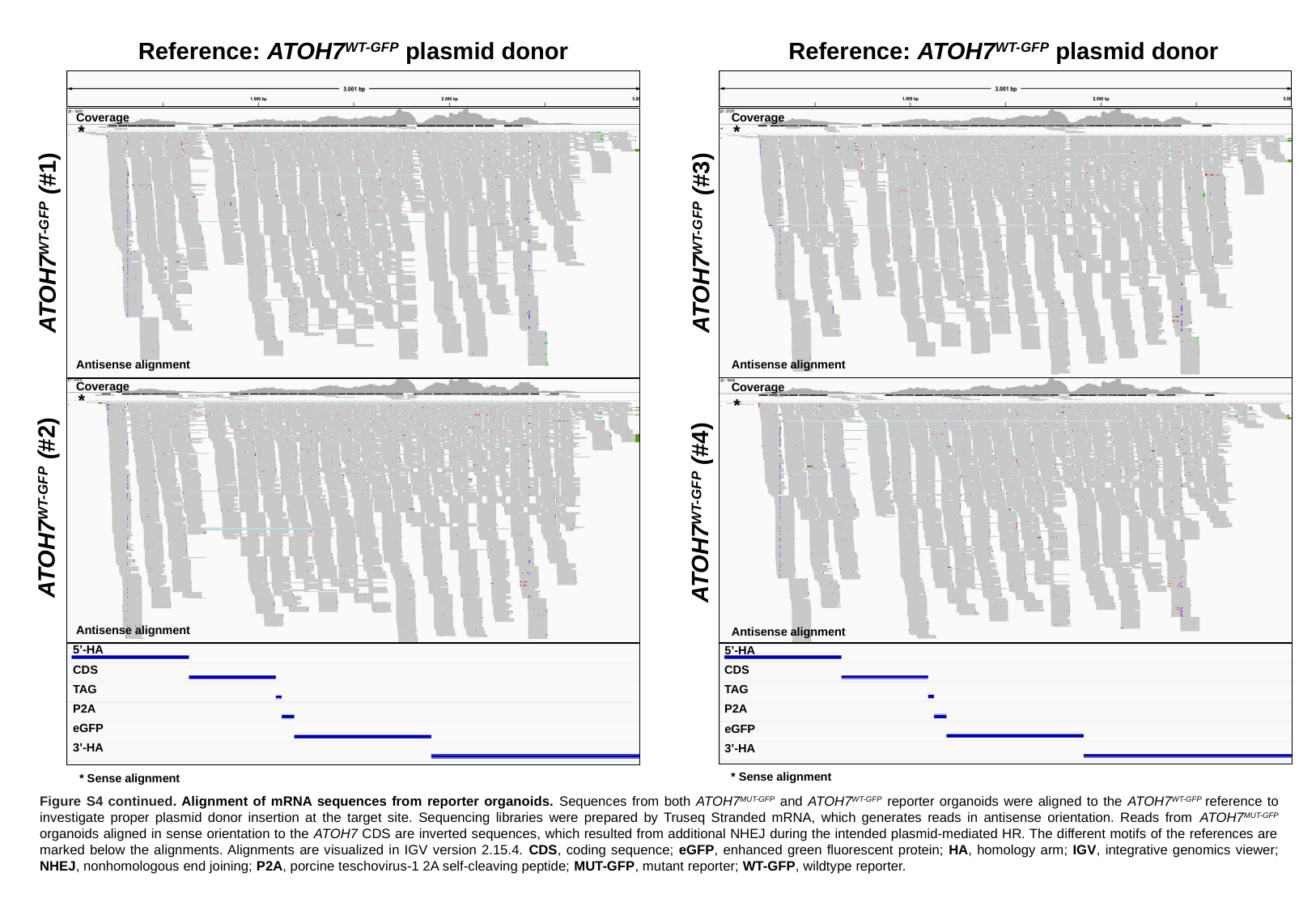

Reference: ATOH7WT-GFP plasmid donor
Reference: ATOH7WT-GFP plasmid donor
Coverage
Coverage
*
*
ATOH7WT-GFP (#1)
ATOH7WT-GFP (#3)
Antisense alignment
Antisense alignment
Coverage
Coverage
*
*
ATOH7WT-GFP (#2)
ATOH7WT-GFP (#4)
Antisense alignment
Antisense alignment
5’-HA
CDS
TAG
P2A
eGFP
3’-HA
5’-HA
CDS
TAG
P2A
eGFP
3’-HA
* Sense alignment
* Sense alignment
Figure S4 continued. Alignment of mRNA sequences from reporter organoids. Sequences from both ATOH7MUT-GFP and ATOH7WT-GFP reporter organoids were aligned to the ATOH7WT-GFP reference to investigate proper plasmid donor insertion at the target site. Sequencing libraries were prepared by Truseq Stranded mRNA, which generates reads in antisense orientation. Reads from ATOH7MUT-GFP organoids aligned in sense orientation to the ATOH7 CDS are inverted sequences, which resulted from additional NHEJ during the intended plasmid-mediated HR. The different motifs of the references are marked below the alignments. Alignments are visualized in IGV version 2.15.4. CDS, coding sequence; eGFP, enhanced green fluorescent protein; HA, homology arm; IGV, integrative genomics viewer; NHEJ, nonhomologous end joining; P2A, porcine teschovirus-1 2A self-cleaving peptide; MUT-GFP, mutant reporter; WT-GFP, wildtype reporter.

## Slide 6
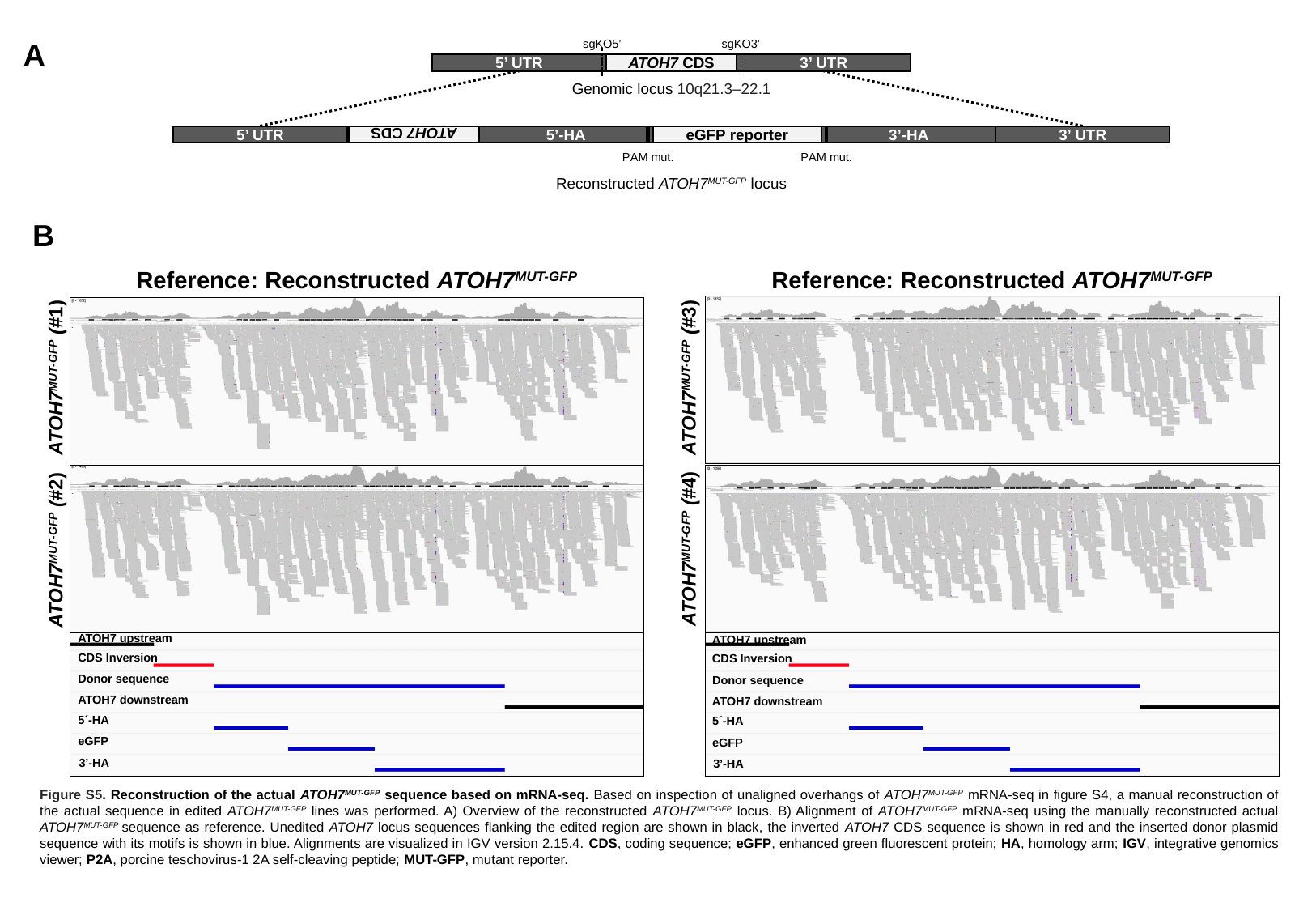

A
sgKO5’
sgKO3’
5’ UTR
ATOH7 CDS
3’ UTR
Genomic locus 10q21.3–22.1
5’ UTR
ATOH7 CDS
5’-HA
eGFP reporter
3’-HA
3’ UTR
PAM mut.
PAM mut.
Reconstructed ATOH7MUT-GFP locus
B
Reference: Reconstructed ATOH7MUT-GFP
Reference: Reconstructed ATOH7MUT-GFP
ATOH7MUT-GFP (#3)
ATOH7MUT-GFP (#1)
ATOH7MUT-GFP (#4)
ATOH7MUT-GFP (#2)
ATOH7 upstream
ATOH7 upstream
CDS Inversion
CDS Inversion
Donor sequence
Donor sequence
ATOH7 downstream
ATOH7 downstream
5´-HA
5´-HA
eGFP
eGFP
3’-HA
3’-HA
Figure S5. Reconstruction of the actual ATOH7MUT-GFP sequence based on mRNA-seq. Based on inspection of unaligned overhangs of ATOH7MUT-GFP mRNA-seq in figure S4, a manual reconstruction of the actual sequence in edited ATOH7MUT-GFP lines was performed. A) Overview of the reconstructed ATOH7MUT-GFP locus. B) Alignment of ATOH7MUT-GFP mRNA-seq using the manually reconstructed actual ATOH7MUT-GFP sequence as reference. Unedited ATOH7 locus sequences flanking the edited region are shown in black, the inverted ATOH7 CDS sequence is shown in red and the inserted donor plasmid sequence with its motifs is shown in blue. Alignments are visualized in IGV version 2.15.4. CDS, coding sequence; eGFP, enhanced green fluorescent protein; HA, homology arm; IGV, integrative genomics viewer; P2A, porcine teschovirus-1 2A self-cleaving peptide; MUT-GFP, mutant reporter.

## Slide 7
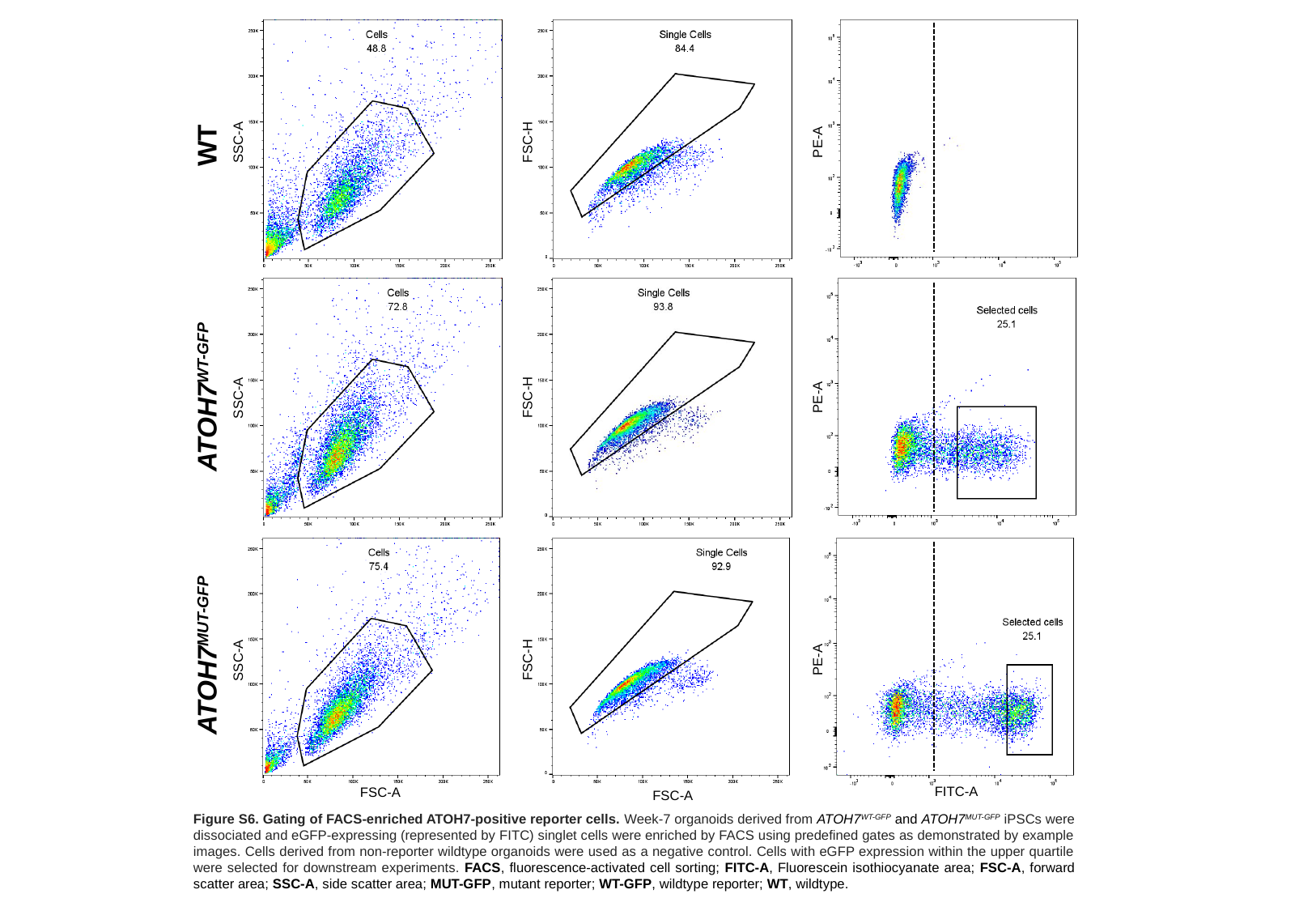

WT
PE-A
FSC-H
SSC-A
ATOH7WT-GFP
PE-A
FSC-H
SSC-A
ATOH7MUT-GFP
PE-A
FSC-H
SSC-A
FITC-A
FSC-A
FSC-A
Figure S6. Gating of FACS-enriched ATOH7-positive reporter cells. Week-7 organoids derived from ATOH7WT-GFP and ATOH7MUT-GFP iPSCs were dissociated and eGFP-expressing (represented by FITC) singlet cells were enriched by FACS using predefined gates as demonstrated by example images. Cells derived from non-reporter wildtype organoids were used as a negative control. Cells with eGFP expression within the upper quartile were selected for downstream experiments. FACS, fluorescence-activated cell sorting; FITC-A, Fluorescein isothiocyanate area; FSC-A, forward scatter area; SSC-A, side scatter area; MUT-GFP, mutant reporter; WT-GFP, wildtype reporter; WT, wildtype.

## Slide 8
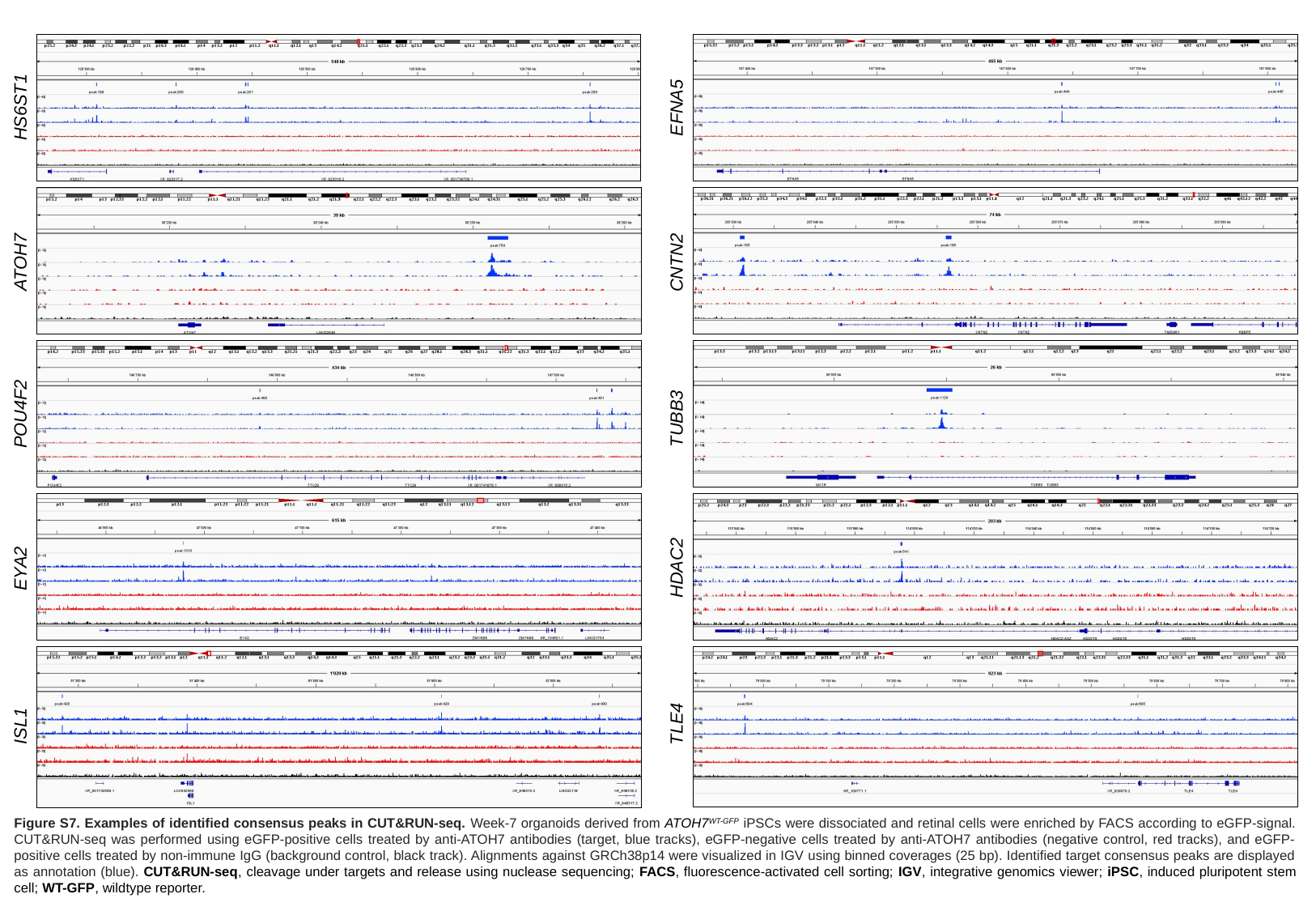

HS6ST1
EFNA5
ATOH7
CNTN2
POU4F2
TUBB3
HDAC2
EYA2
TLE4
ISL1
Figure S7. Examples of identified consensus peaks in CUT&RUN-seq. Week-7 organoids derived from ATOH7WT-GFP iPSCs were dissociated and retinal cells were enriched by FACS according to eGFP-signal. CUT&RUN-seq was performed using eGFP-positive cells treated by anti-ATOH7 antibodies (target, blue tracks), eGFP-negative cells treated by anti-ATOH7 antibodies (negative control, red tracks), and eGFP-positive cells treated by non-immune IgG (background control, black track). Alignments against GRCh38p14 were visualized in IGV using binned coverages (25 bp). Identified target consensus peaks are displayed as annotation (blue). CUT&RUN-seq, cleavage under targets and release using nuclease sequencing; FACS, fluorescence-activated cell sorting; IGV, integrative genomics viewer; iPSC, induced pluripotent stem cell; WT-GFP, wildtype reporter.

## Slide 9
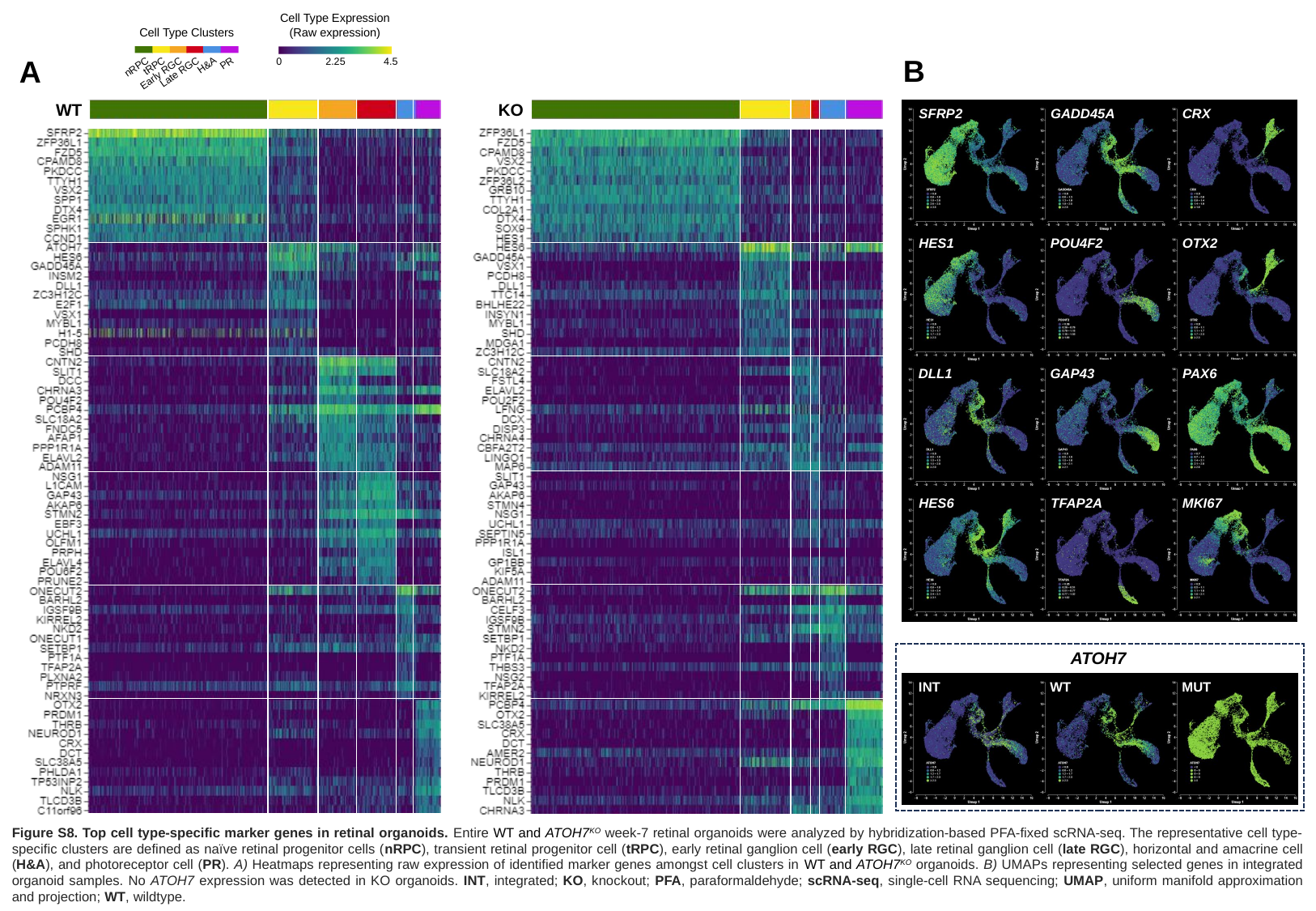

Cell Type Expression
(Raw expression)
0
2.25
4.5
Cell Type Clusters
PR
H&A
tRPC
nRPC
Late RGC
Early RGC
B
A
WT
KO
SFRP2
GADD45A
CRX
HES1
POU4F2
OTX2
DLL1
GAP43
PAX6
HES6
TFAP2A
MKI67
ATOH7
INT
WT
MUT
Figure S8. Top cell type-specific marker genes in retinal organoids. Entire WT and ATOH7KO week-7 retinal organoids were analyzed by hybridization-based PFA-fixed scRNA-seq. The representative cell type-specific clusters are defined as naïve retinal progenitor cells (nRPC), transient retinal progenitor cell (tRPC), early retinal ganglion cell (early RGC), late retinal ganglion cell (late RGC), horizontal and amacrine cell (H&A), and photoreceptor cell (PR). A) Heatmaps representing raw expression of identified marker genes amongst cell clusters in WT and ATOH7KO organoids. B) UMAPs representing selected genes in integrated organoid samples. No ATOH7 expression was detected in KO organoids. INT, integrated; KO, knockout; PFA, paraformaldehyde; scRNA-seq, single-cell RNA sequencing; UMAP, uniform manifold approximation and projection; WT, wildtype.

## Slide 10
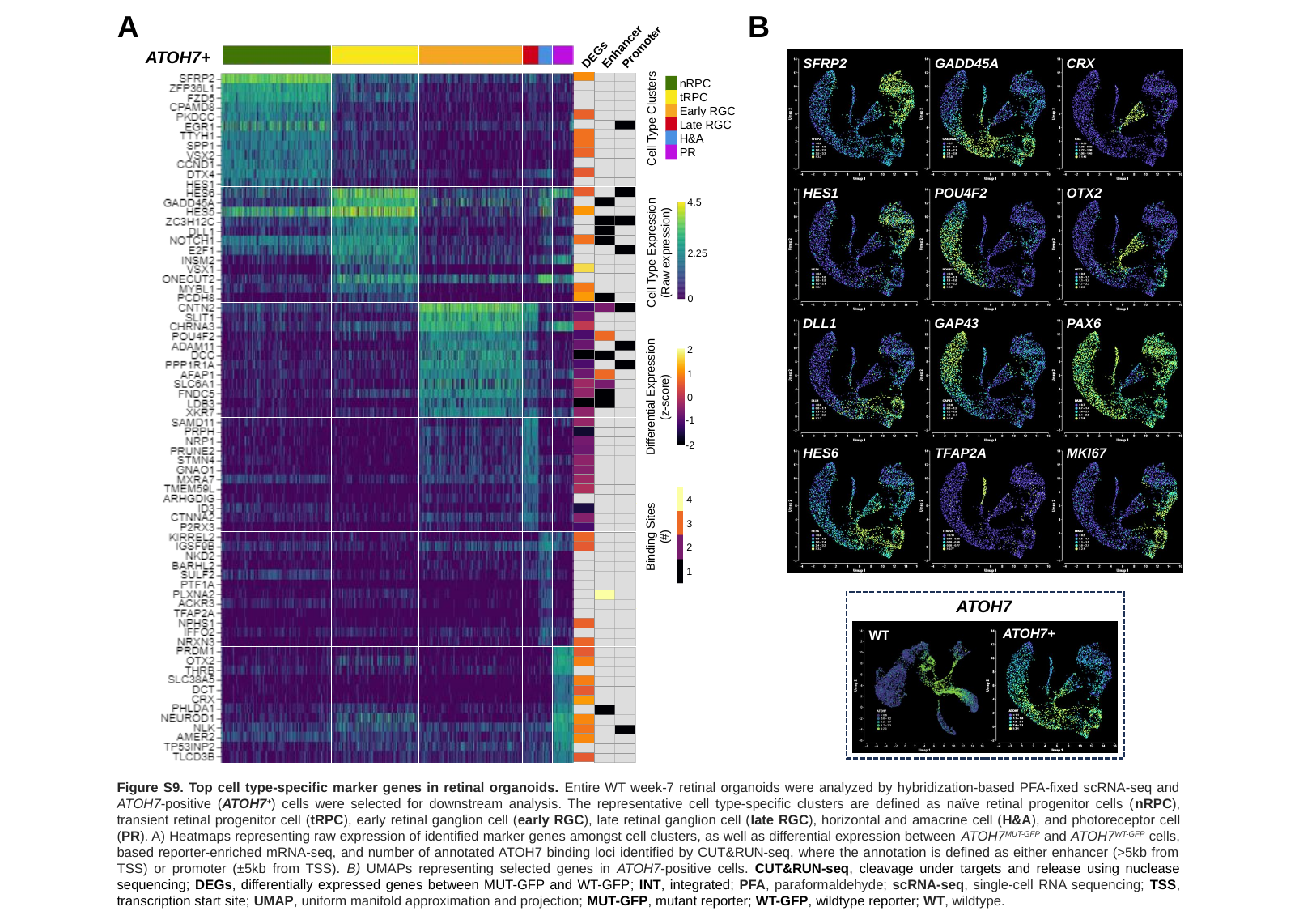

A
B
ATOH7+
SFRP2
GADD45A
CRX
nRPC
tRPC
Early RGC
Late RGC
H&A
PR
Cell Type Clusters
HES1
POU4F2
OTX2
Cell Type Expression
(Raw expression)
0
4.5
2.25
DLL1
GAP43
PAX6
Differential Expression (z-score)
-2
-1
0
1
2
HES6
TFAP2A
MKI67
Binding Sites
(#)
1
2
4
3
ATOH7
ATOH7+
WT
Figure S9. Top cell type-specific marker genes in retinal organoids. Entire WT week-7 retinal organoids were analyzed by hybridization-based PFA-fixed scRNA-seq and ATOH7-positive (ATOH7+) cells were selected for downstream analysis. The representative cell type-specific clusters are defined as naïve retinal progenitor cells (nRPC), transient retinal progenitor cell (tRPC), early retinal ganglion cell (early RGC), late retinal ganglion cell (late RGC), horizontal and amacrine cell (H&A), and photoreceptor cell (PR). A) Heatmaps representing raw expression of identified marker genes amongst cell clusters, as well as differential expression between ATOH7MUT-GFP and ATOH7WT-GFP cells, based reporter-enriched mRNA-seq, and number of annotated ATOH7 binding loci identified by CUT&RUN-seq, where the annotation is defined as either enhancer (>5kb from TSS) or promoter (±5kb from TSS). B) UMAPs representing selected genes in ATOH7-positive cells. CUT&RUN-seq, cleavage under targets and release using nuclease sequencing; DEGs, differentially expressed genes between MUT-GFP and WT-GFP; INT, integrated; PFA, paraformaldehyde; scRNA-seq, single-cell RNA sequencing; TSS, transcription start site; UMAP, uniform manifold approximation and projection; MUT-GFP, mutant reporter; WT-GFP, wildtype reporter; WT, wildtype.
DEGs
Promoter
Enhancer

## Slide 11
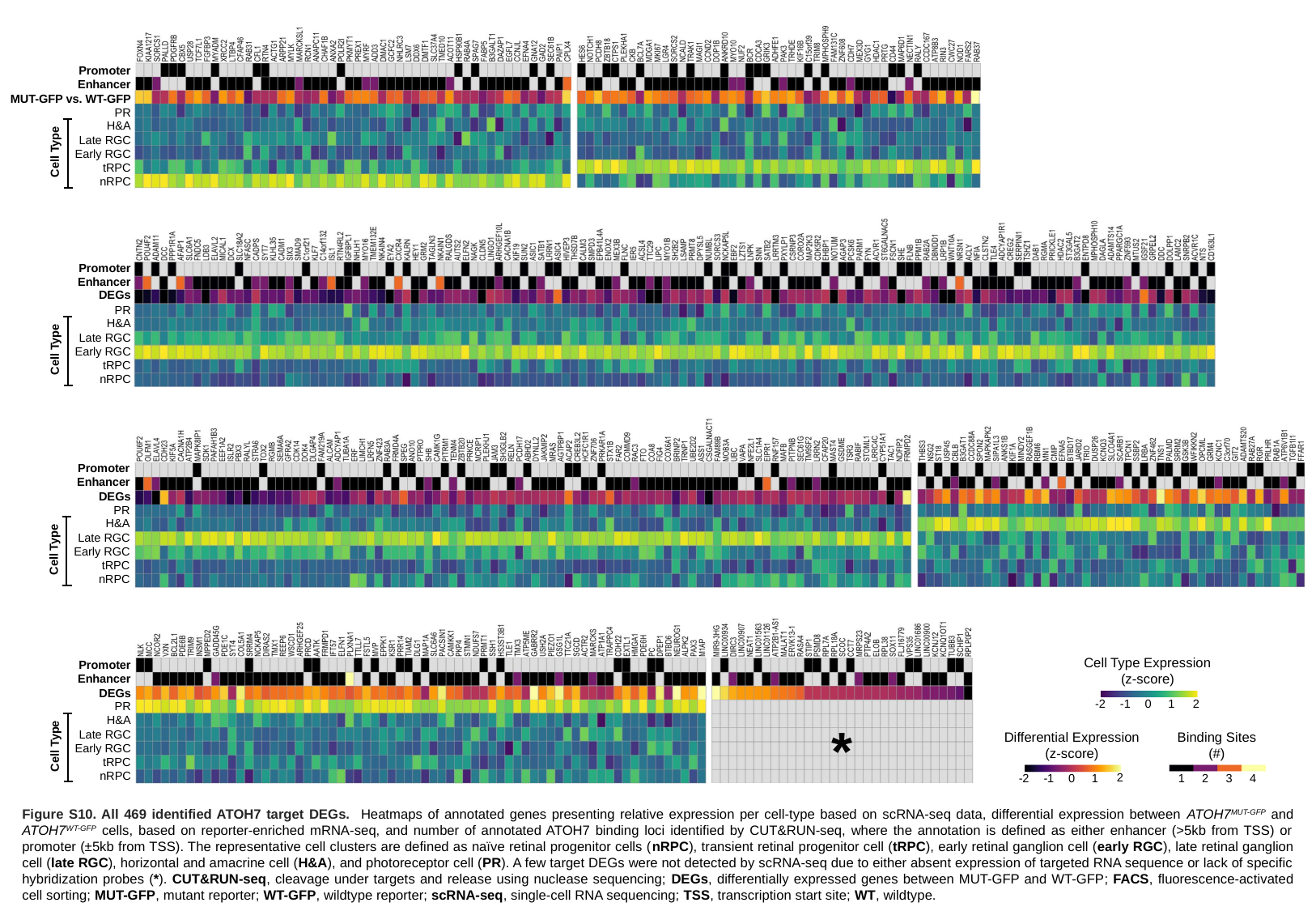

Promoter
Enhancer
MUT-GFP vs. WT-GFP
PR
H&A
Late RGC
Cell Type
Early RGC
tRPC
nRPC
Promoter
Enhancer
DEGs
PR
H&A
Late RGC
Cell Type
Early RGC
tRPC
nRPC
Promoter
Enhancer
DEGs
PR
H&A
Late RGC
Cell Type
Early RGC
tRPC
nRPC
Cell Type Expression
(z-score)
-2
-1
0
1
2
Binding Sites
(#)
1
2
3
4
Differential Expression
(z-score)
2
-2
-1
0
1
Promoter
Enhancer
DEGs
PR
H&A
Late RGC
Cell Type
Early RGC
tRPC
nRPC
*
Figure S10. All 469 identified ATOH7 target DEGs. Heatmaps of annotated genes presenting relative expression per cell-type based on scRNA-seq data, differential expression between ATOH7MUT-GFP and ATOH7WT-GFP cells, based on reporter-enriched mRNA-seq, and number of annotated ATOH7 binding loci identified by CUT&RUN-seq, where the annotation is defined as either enhancer (>5kb from TSS) or promoter (±5kb from TSS). The representative cell clusters are defined as naïve retinal progenitor cells (nRPC), transient retinal progenitor cell (tRPC), early retinal ganglion cell (early RGC), late retinal ganglion cell (late RGC), horizontal and amacrine cell (H&A), and photoreceptor cell (PR). A few target DEGs were not detected by scRNA-seq due to either absent expression of targeted RNA sequence or lack of specific hybridization probes (*). CUT&RUN-seq, cleavage under targets and release using nuclease sequencing; DEGs, differentially expressed genes between MUT-GFP and WT-GFP; FACS, fluorescence-activated cell sorting; MUT-GFP, mutant reporter; WT-GFP, wildtype reporter; scRNA-seq, single-cell RNA sequencing; TSS, transcription start site; WT, wildtype.

## Slide 12
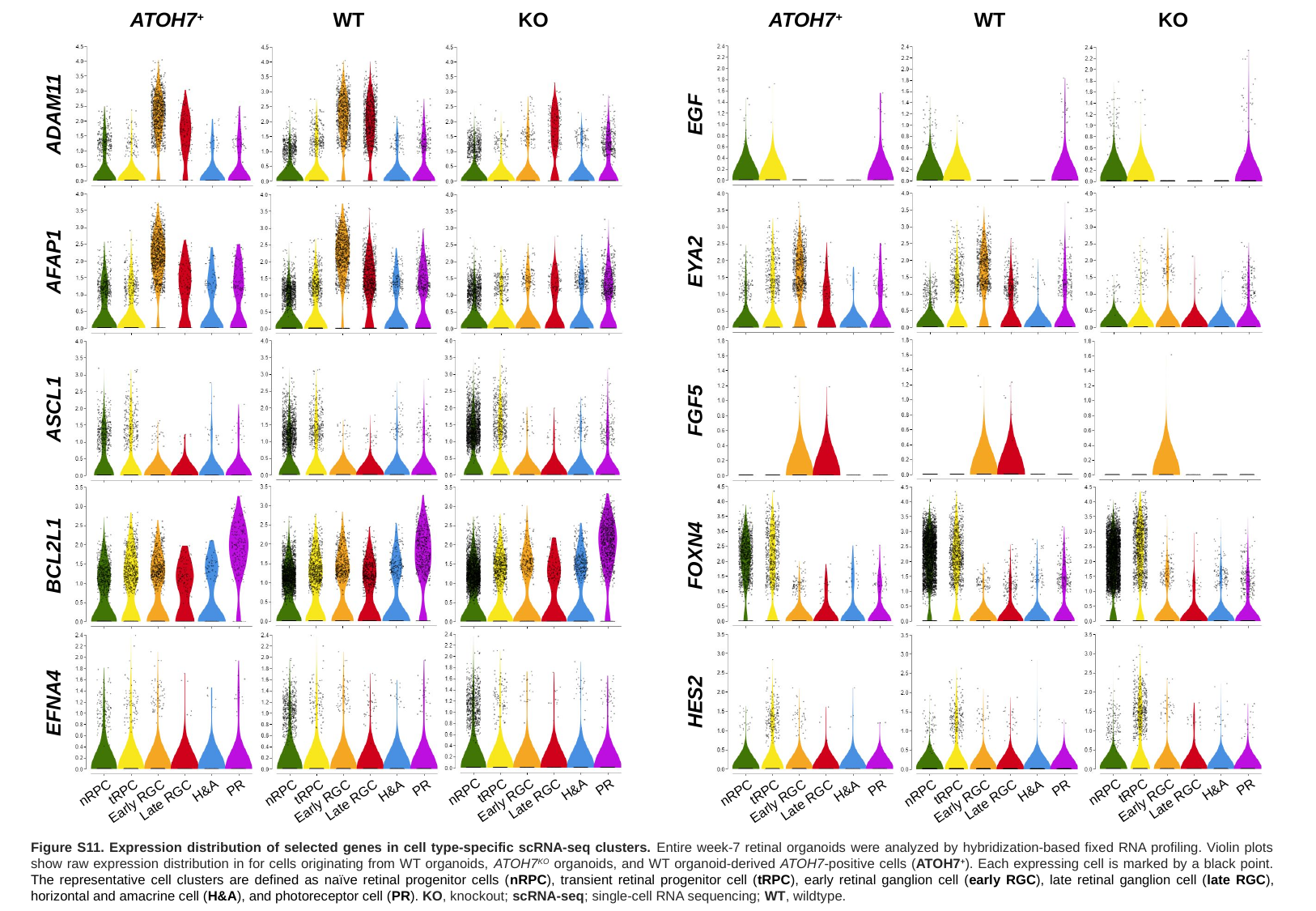

ATOH7+
WT
KO
ATOH7+
WT
KO
ADAM11
EGF
AFAP1
EYA2
ASCL1
FGF5
BCL2L1
FOXN4
HES2
EFNA4
PR
PR
PR
PR
PR
PR
H&A
H&A
H&A
H&A
H&A
H&A
tRPC
tRPC
nRPC
nRPC
tRPC
tRPC
tRPC
tRPC
nRPC
nRPC
nRPC
nRPC
Late RGC
Late RGC
Late RGC
Late RGC
Late RGC
Late RGC
Early RGC
Early RGC
Early RGC
Early RGC
Early RGC
Early RGC
Figure S11. Expression distribution of selected genes in cell type-specific scRNA-seq clusters. Entire week-7 retinal organoids were analyzed by hybridization-based fixed RNA profiling. Violin plots show raw expression distribution in for cells originating from WT organoids, ATOH7KO organoids, and WT organoid-derived ATOH7-positive cells (ATOH7+). Each expressing cell is marked by a black point. The representative cell clusters are defined as naïve retinal progenitor cells (nRPC), transient retinal progenitor cell (tRPC), early retinal ganglion cell (early RGC), late retinal ganglion cell (late RGC), horizontal and amacrine cell (H&A), and photoreceptor cell (PR). KO, knockout; scRNA-seq; single-cell RNA sequencing; WT, wildtype.

## Slide 13
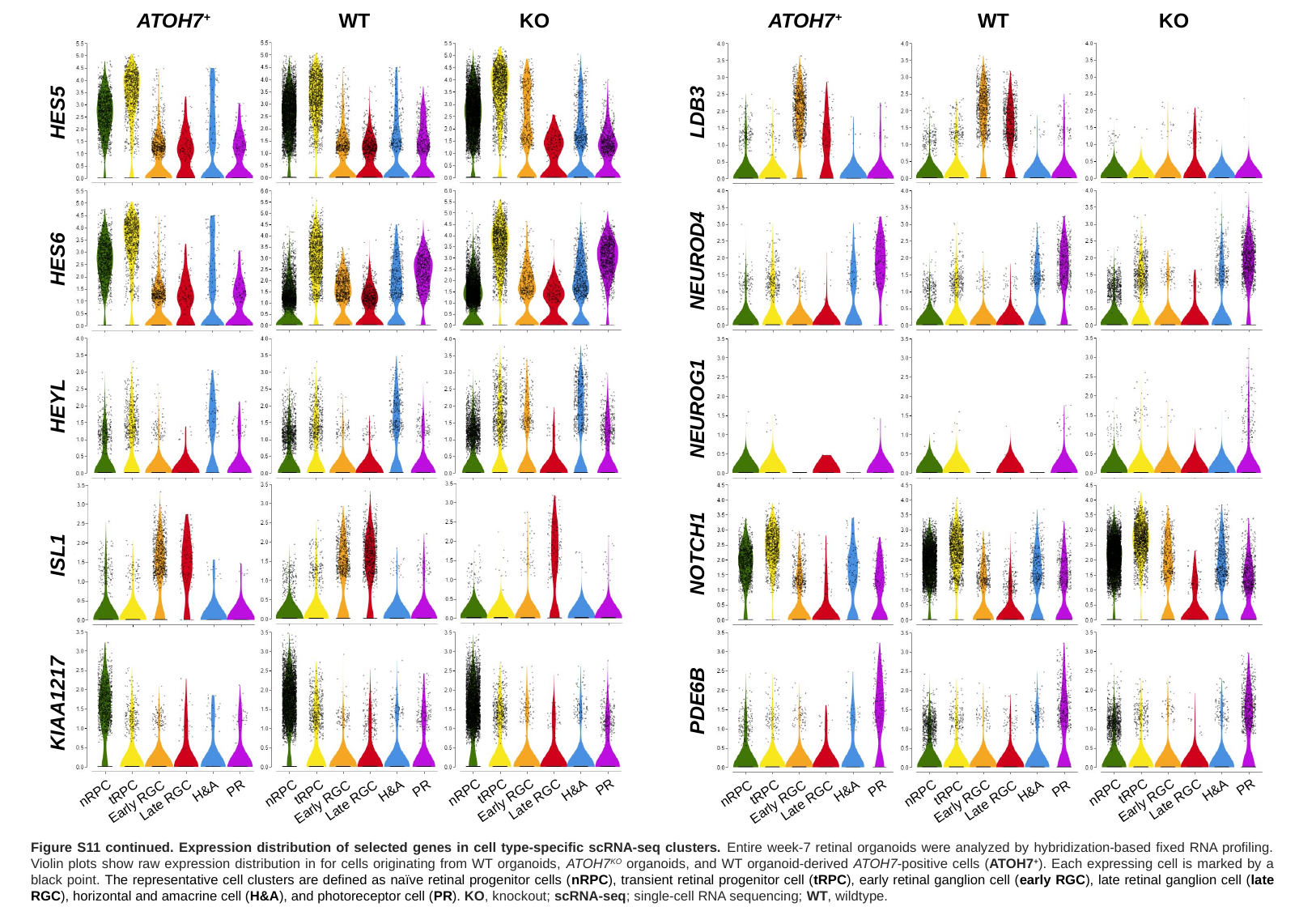

ATOH7+
WT
KO
ATOH7+
WT
KO
HES5
LDB3
HES6
NEUROD4
HEYL
NEUROG1
NOTCH1
ISL1
PDE6B
KIAA1217
PR
PR
PR
PR
PR
PR
H&A
H&A
H&A
H&A
H&A
H&A
tRPC
tRPC
nRPC
nRPC
tRPC
tRPC
tRPC
tRPC
nRPC
nRPC
nRPC
nRPC
Late RGC
Late RGC
Late RGC
Late RGC
Late RGC
Late RGC
Early RGC
Early RGC
Early RGC
Early RGC
Early RGC
Early RGC
Figure S11 continued. Expression distribution of selected genes in cell type-specific scRNA-seq clusters. Entire week-7 retinal organoids were analyzed by hybridization-based fixed RNA profiling. Violin plots show raw expression distribution in for cells originating from WT organoids, ATOH7KO organoids, and WT organoid-derived ATOH7-positive cells (ATOH7+). Each expressing cell is marked by a black point. The representative cell clusters are defined as naïve retinal progenitor cells (nRPC), transient retinal progenitor cell (tRPC), early retinal ganglion cell (early RGC), late retinal ganglion cell (late RGC), horizontal and amacrine cell (H&A), and photoreceptor cell (PR). KO, knockout; scRNA-seq; single-cell RNA sequencing; WT, wildtype.

## Slide 14
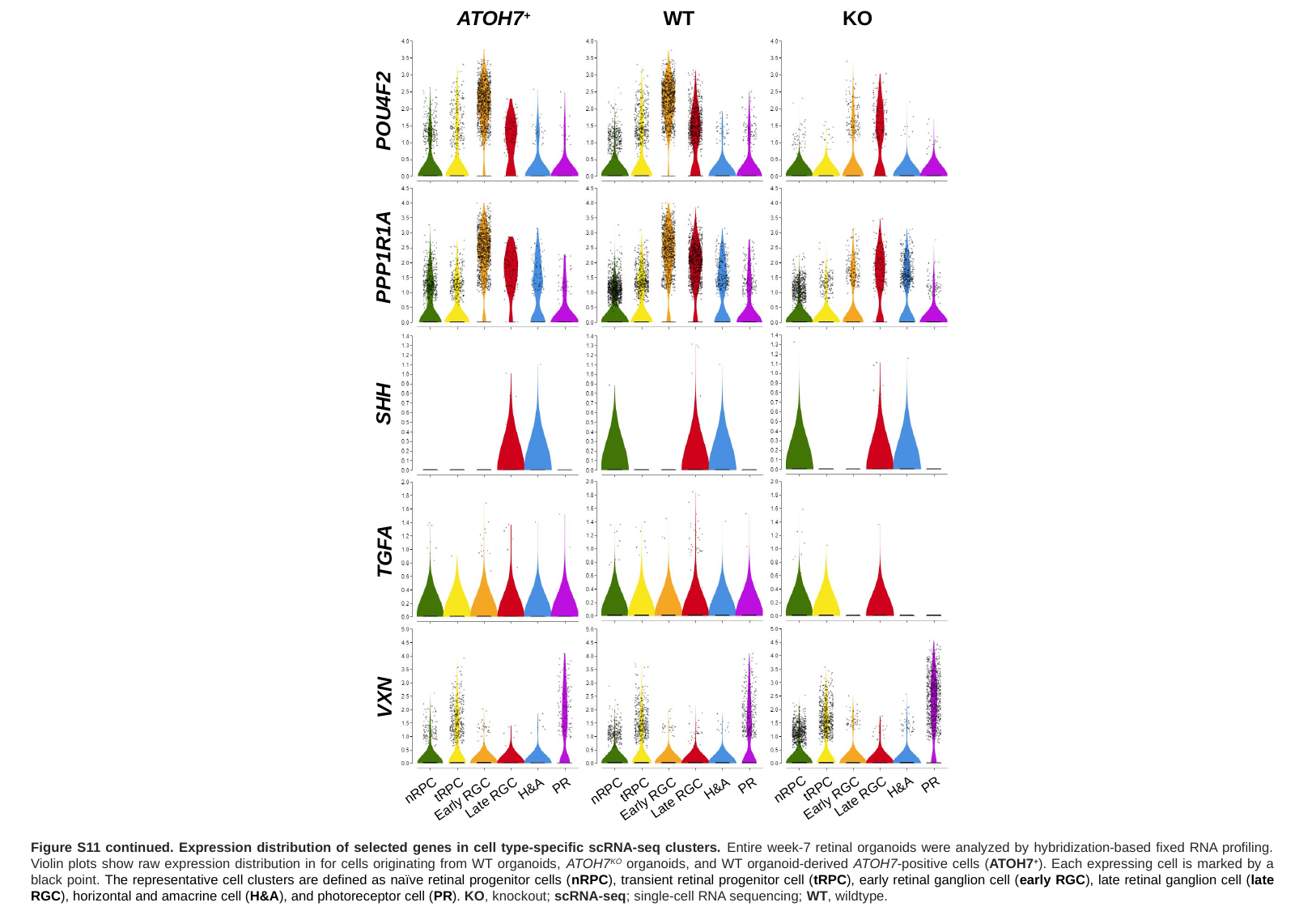

ATOH7+
WT
KO
POU4F2
PPP1R1A
SHH
TGFA
VXN
PR
PR
PR
H&A
H&A
H&A
tRPC
nRPC
tRPC
tRPC
nRPC
nRPC
Late RGC
Late RGC
Late RGC
Early RGC
Early RGC
Early RGC
Figure S11 continued. Expression distribution of selected genes in cell type-specific scRNA-seq clusters. Entire week-7 retinal organoids were analyzed by hybridization-based fixed RNA profiling. Violin plots show raw expression distribution in for cells originating from WT organoids, ATOH7KO organoids, and WT organoid-derived ATOH7-positive cells (ATOH7+). Each expressing cell is marked by a black point. The representative cell clusters are defined as naïve retinal progenitor cells (nRPC), transient retinal progenitor cell (tRPC), early retinal ganglion cell (early RGC), late retinal ganglion cell (late RGC), horizontal and amacrine cell (H&A), and photoreceptor cell (PR). KO, knockout; scRNA-seq; single-cell RNA sequencing; WT, wildtype.

## Slide 15
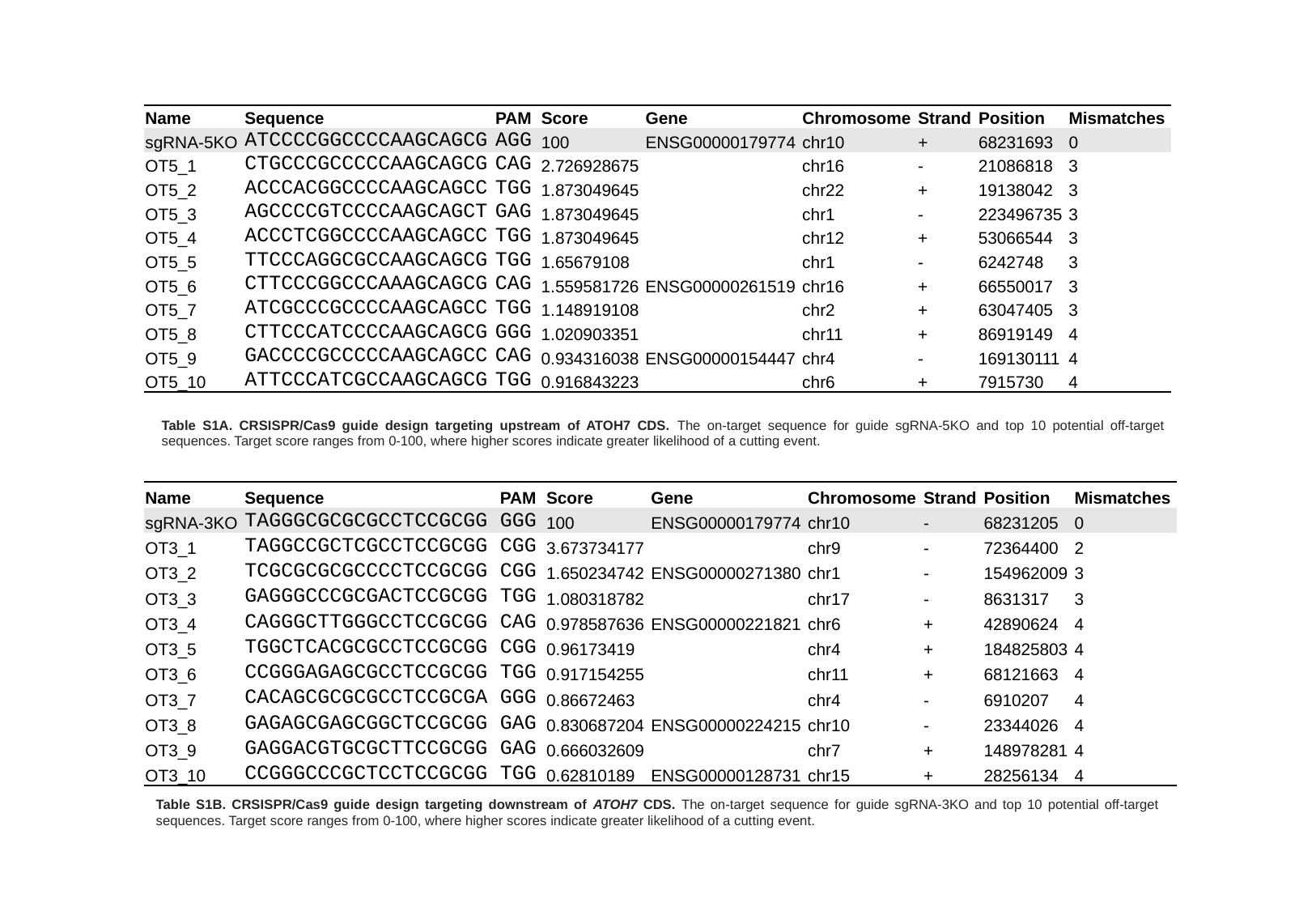

| Name | Sequence | PAM | Score | Gene | Chromosome | Strand | Position | Mismatches |
| --- | --- | --- | --- | --- | --- | --- | --- | --- |
| sgRNA-5KO | ATCCCCGGCCCCAAGCAGCG | AGG | 100 | ENSG00000179774 | chr10 | + | 68231693 | 0 |
| OT5\_1 | CTGCCCGCCCCCAAGCAGCG | CAG | 2.726928675 | | chr16 | - | 21086818 | 3 |
| OT5\_2 | ACCCACGGCCCCAAGCAGCC | TGG | 1.873049645 | | chr22 | + | 19138042 | 3 |
| OT5\_3 | AGCCCCGTCCCCAAGCAGCT | GAG | 1.873049645 | | chr1 | - | 223496735 | 3 |
| OT5\_4 | ACCCTCGGCCCCAAGCAGCC | TGG | 1.873049645 | | chr12 | + | 53066544 | 3 |
| OT5\_5 | TTCCCAGGCGCCAAGCAGCG | TGG | 1.65679108 | | chr1 | - | 6242748 | 3 |
| OT5\_6 | CTTCCCGGCCCAAAGCAGCG | CAG | 1.559581726 | ENSG00000261519 | chr16 | + | 66550017 | 3 |
| OT5\_7 | ATCGCCCGCCCCAAGCAGCC | TGG | 1.148919108 | | chr2 | + | 63047405 | 3 |
| OT5\_8 | CTTCCCATCCCCAAGCAGCG | GGG | 1.020903351 | | chr11 | + | 86919149 | 4 |
| OT5\_9 | GACCCCGCCCCCAAGCAGCC | CAG | 0.934316038 | ENSG00000154447 | chr4 | - | 169130111 | 4 |
| OT5\_10 | ATTCCCATCGCCAAGCAGCG | TGG | 0.916843223 | | chr6 | + | 7915730 | 4 |
Table S1A. CRSISPR/Cas9 guide design targeting upstream of ATOH7 CDS. The on-target sequence for guide sgRNA-5KO and top 10 potential off-target sequences. Target score ranges from 0-100, where higher scores indicate greater likelihood of a cutting event.
| Name | Sequence | PAM | Score | Gene | Chromosome | Strand | Position | Mismatches |
| --- | --- | --- | --- | --- | --- | --- | --- | --- |
| sgRNA-3KO | TAGGGCGCGCGCCTCCGCGG | GGG | 100 | ENSG00000179774 | chr10 | - | 68231205 | 0 |
| OT3\_1 | TAGGCCGCTCGCCTCCGCGG | CGG | 3.673734177 | | chr9 | - | 72364400 | 2 |
| OT3\_2 | TCGCGCGCGCCCCTCCGCGG | CGG | 1.650234742 | ENSG00000271380 | chr1 | - | 154962009 | 3 |
| OT3\_3 | GAGGGCCCGCGACTCCGCGG | TGG | 1.080318782 | | chr17 | - | 8631317 | 3 |
| OT3\_4 | CAGGGCTTGGGCCTCCGCGG | CAG | 0.978587636 | ENSG00000221821 | chr6 | + | 42890624 | 4 |
| OT3\_5 | TGGCTCACGCGCCTCCGCGG | CGG | 0.96173419 | | chr4 | + | 184825803 | 4 |
| OT3\_6 | CCGGGAGAGCGCCTCCGCGG | TGG | 0.917154255 | | chr11 | + | 68121663 | 4 |
| OT3\_7 | CACAGCGCGCGCCTCCGCGA | GGG | 0.86672463 | | chr4 | - | 6910207 | 4 |
| OT3\_8 | GAGAGCGAGCGGCTCCGCGG | GAG | 0.830687204 | ENSG00000224215 | chr10 | - | 23344026 | 4 |
| OT3\_9 | GAGGACGTGCGCTTCCGCGG | GAG | 0.666032609 | | chr7 | + | 148978281 | 4 |
| OT3\_10 | CCGGGCCCGCTCCTCCGCGG | TGG | 0.62810189 | ENSG00000128731 | chr15 | + | 28256134 | 4 |
Table S1B. CRSISPR/Cas9 guide design targeting downstream of ATOH7 CDS. The on-target sequence for guide sgRNA-3KO and top 10 potential off-target sequences. Target score ranges from 0-100, where higher scores indicate greater likelihood of a cutting event.

## Slide 16
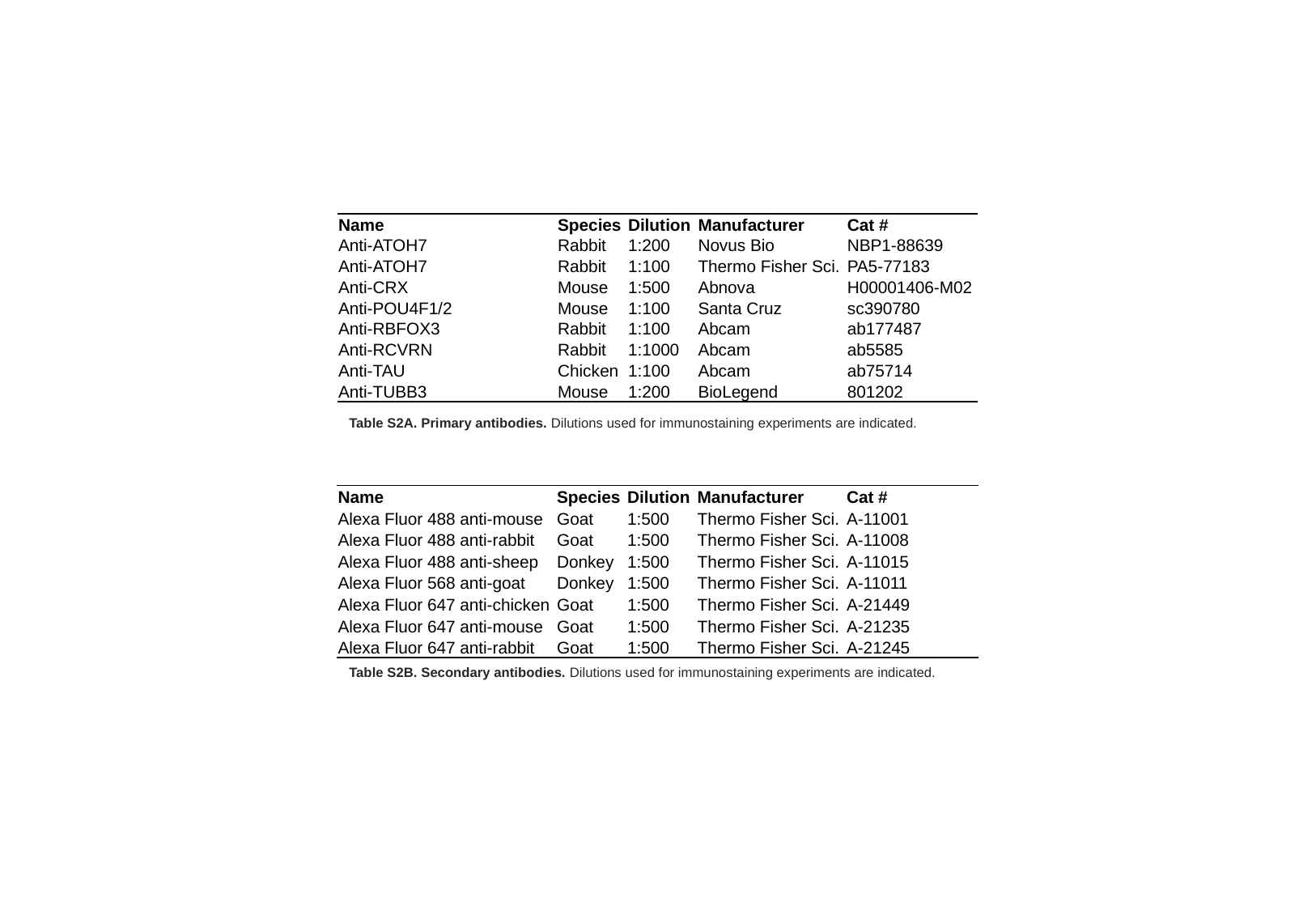

| Name | Species | Dilution | Manufacturer | Cat # |
| --- | --- | --- | --- | --- |
| Anti-ATOH7 | Rabbit | 1:200 | Novus Bio | NBP1-88639 |
| Anti-ATOH7 | Rabbit | 1:100 | Thermo Fisher Sci. | PA5-77183 |
| Anti-CRX | Mouse | 1:500 | Abnova | H00001406-M02 |
| Anti-POU4F1/2 | Mouse | 1:100 | Santa Cruz | sc390780 |
| Anti-RBFOX3 | Rabbit | 1:100 | Abcam | ab177487 |
| Anti-RCVRN | Rabbit | 1:1000 | Abcam | ab5585 |
| Anti-TAU | Chicken | 1:100 | Abcam | ab75714 |
| Anti-TUBB3 | Mouse | 1:200 | BioLegend | 801202 |
Table S2A. Primary antibodies. Dilutions used for immunostaining experiments are indicated.
| Name | Species | Dilution | Manufacturer | Cat # |
| --- | --- | --- | --- | --- |
| Alexa Fluor 488 anti-mouse | Goat | 1:500 | Thermo Fisher Sci. | A-11001 |
| Alexa Fluor 488 anti-rabbit | Goat | 1:500 | Thermo Fisher Sci. | A-11008 |
| Alexa Fluor 488 anti-sheep | Donkey | 1:500 | Thermo Fisher Sci. | A-11015 |
| Alexa Fluor 568 anti-goat | Donkey | 1:500 | Thermo Fisher Sci. | A-11011 |
| Alexa Fluor 647 anti-chicken | Goat | 1:500 | Thermo Fisher Sci. | A-21449 |
| Alexa Fluor 647 anti-mouse | Goat | 1:500 | Thermo Fisher Sci. | A-21235 |
| Alexa Fluor 647 anti-rabbit | Goat | 1:500 | Thermo Fisher Sci. | A-21245 |
Table S2B. Secondary antibodies. Dilutions used for immunostaining experiments are indicated.

## Slide 17
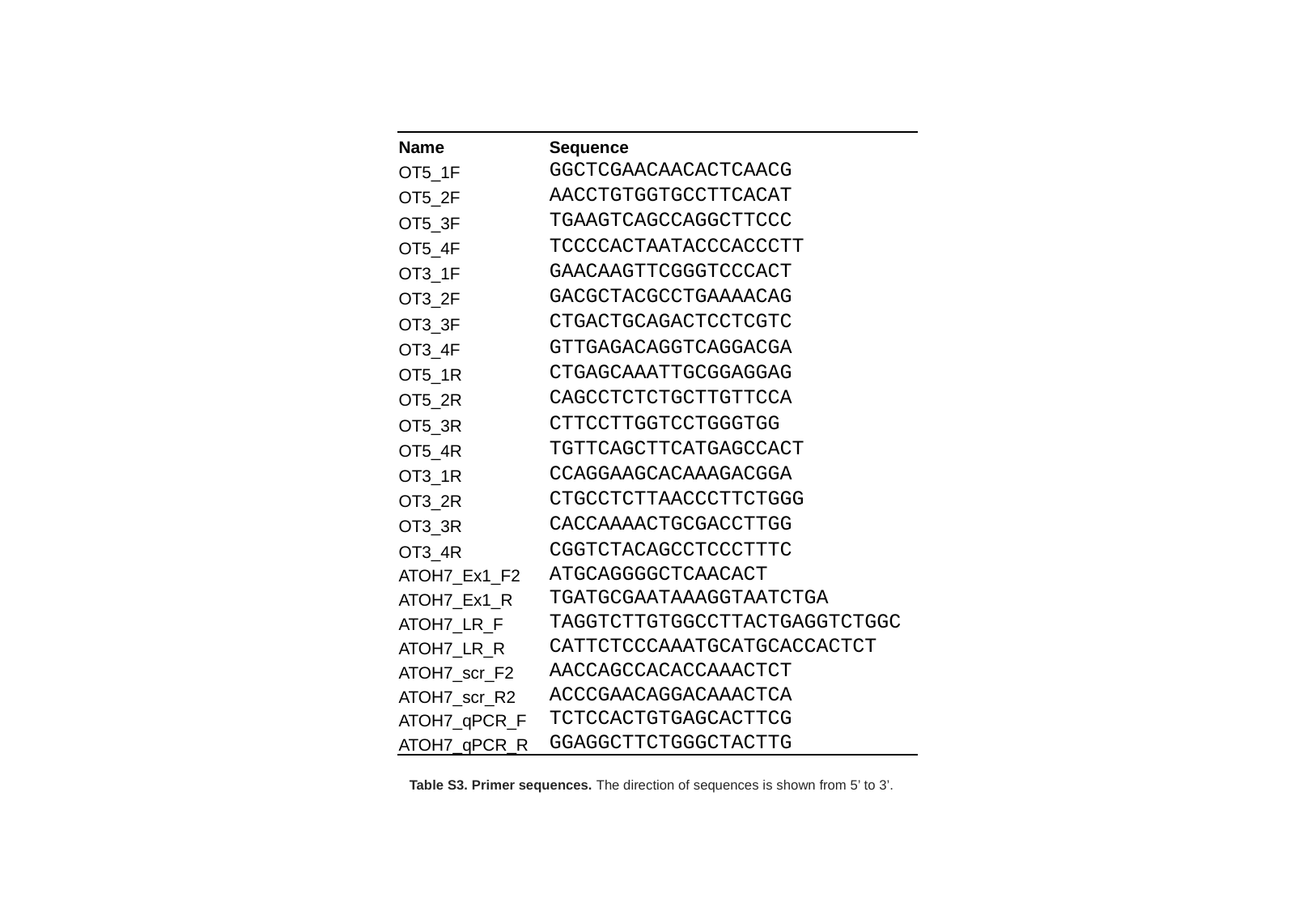

| Name | Sequence |
| --- | --- |
| OT5\_1F | GGCTCGAACAACACTCAACG |
| OT5\_2F | AACCTGTGGTGCCTTCACAT |
| OT5\_3F | TGAAGTCAGCCAGGCTTCCC |
| OT5\_4F | TCCCCACTAATACCCACCCTT |
| OT3\_1F | GAACAAGTTCGGGTCCCACT |
| OT3\_2F | GACGCTACGCCTGAAAACAG |
| OT3\_3F | CTGACTGCAGACTCCTCGTC |
| OT3\_4F | GTTGAGACAGGTCAGGACGA |
| OT5\_1R | CTGAGCAAATTGCGGAGGAG |
| OT5\_2R | CAGCCTCTCTGCTTGTTCCA |
| OT5\_3R | CTTCCTTGGTCCTGGGTGG |
| OT5\_4R | TGTTCAGCTTCATGAGCCACT |
| OT3\_1R | CCAGGAAGCACAAAGACGGA |
| OT3\_2R | CTGCCTCTTAACCCTTCTGGG |
| OT3\_3R | CACCAAAACTGCGACCTTGG |
| OT3\_4R | CGGTCTACAGCCTCCCTTTC |
| ATOH7\_Ex1\_F2 | ATGCAGGGGCTCAACACT |
| ATOH7\_Ex1\_R | TGATGCGAATAAAGGTAATCTGA |
| ATOH7\_LR\_F | TAGGTCTTGTGGCCTTACTGAGGTCTGGC |
| ATOH7\_LR\_R | CATTCTCCCAAATGCATGCACCACTCT |
| ATOH7\_scr\_F2 | AACCAGCCACACCAAACTCT |
| ATOH7\_scr\_R2 | ACCCGAACAGGACAAACTCA |
| ATOH7\_qPCR\_F | TCTCCACTGTGAGCACTTCG |
| ATOH7\_qPCR\_R | GGAGGCTTCTGGGCTACTTG |
Table S3. Primer sequences. The direction of sequences is shown from 5’ to 3’.
